# Landscape Genetics of Leaf-Toed Geckos in the Tropical Dry Forest of Northern Mexico

**DOI:** 10.1371/journal.pone.0057433

**Published:** 2013-02-25

**Authors:** Christopher Blair, Victor H. Jiménez Arcos, Fausto R. Mendez de la Cruz, Robert W. Murphy

**Affiliations:** 1 Department of Ecology and Evolutionary Biology, University of Toronto, Toronto, Ontario, Canada; 2 Department of Natural History, Royal Ontario Museum, Toronto, Ontario, Canada; 3 Laboratorio de Herpetología, Instituto de Biología, Universidad Nacional Autónoma de México, Mexico City, México; 4 State Key Laboratory of Genetic Resources and Evolution, Kunming Institute of Zoology, the Chinese Academy of Sciences, Kunming, China; University of Plymouth, United Kingdom

## Abstract

Habitat fragmentation due to both natural and anthropogenic forces continues to threaten the evolution and maintenance of biological diversity. This is of particular concern in tropical regions that are experiencing elevated rates of habitat loss. Although less well-studied than tropical rain forests, tropical dry forests (TDF) contain an enormous diversity of species and continue to be threatened by anthropogenic activities including grazing and agriculture. However, little is known about the processes that shape genetic connectivity in species inhabiting TDF ecosystems. We adopt a landscape genetic approach to understanding functional connectivity for leaf-toed geckos (*Phyllodactylus tuberculosus*) at multiple sites near the northernmost limit of this ecosystem at Alamos, Sonora, Mexico. Traditional analyses of population genetics are combined with multivariate GIS-based landscape analyses to test hypotheses on the potential drivers of spatial genetic variation. Moderate levels of within-population diversity and substantial levels of population differentiation are revealed by *F*_ST_ and *D*_est_. Analyses using structure suggest the occurrence of from 2 to 9 genetic clusters depending on the model used. Landscape genetic analysis suggests that forest cover, stream connectivity, undisturbed habitat, slope, and minimum temperature of the coldest period explain more genetic variation than do simple Euclidean distances. Additional landscape genetic studies throughout TDF habitat are required to understand species-specific responses to landscape and climate change and to identify common drivers. We urge researchers interested in using multivariate distance methods to test for, and report, significant correlations among predictor matrices that can impact results, particularly when adopting least-cost path approaches. Further investigation into the use of information theoretic approaches for model selection is also warranted.

## Introduction

Dispersal is a fundamental process that can greatly influence ecological and demographic trajectories within and between subpopulations [1]. For example, dispersal often leads to gene flow, the transfer of genetic information from one population to another [2], [3]. Maintaining adequate rates of gene flow is often beneficial because populations experiencing little gene flow are susceptible to a loss of genetic diversity due to inbreeding and drift [2], [4], [5]. A lack of genetic diversity may also make it difficult for populations to adapt to changing environmental conditions that may lead to local extinction [6], [7].

Differences in dispersal rates can result from factors including species-specific philopatry, intra- and interspecific interactions, predation, physiological tolerances, and simple geographic (Euclidean) distance [8]. Landscape heterogeneity often plays a substantial role in the ability and/or choice of an organism to disperse or not [9]–[11]. Both natural and anthropogenic habitat fragmentation can detrimentally affect the connectivity and persistence of populations [12]–[15]. Landscape genetics seeks to explicitly quantify the influence of landscape and environmental variables on microevolutionary processes such as gene flow and natural selection [16], [17]. The approach extends traditional population genetic studies by explaining the spatial distribution of genetic variation using components of the landscape. This particularly powerful approach to studying fine-scale population structure and its application is meeting with much success [18]. By combining rapidly evolving molecular markers such as microsatellites with novel approaches to statistical analysis, landscape genetics identifies a suite of environmental variables likely to influence population genetic structure [19]–[21]. Identifying the landscape components facilitating or constraining gene flow can aid in delimiting areas for conservation [22], for example, by designing corridors that maximize functional connectivity [23], [24]. Today, few landscape genetic studies focus on tropical areas [18], which harbour the majority of species [25]. Compared to temperate localities, relatively little is known about the processes influencing functional connectivity in species inhabiting this mega-diverse region.

Tropical deciduous or dry forests (TDF), which also occur in the Neotropics, are a major biodiversity hotspot [25]. They form a semi-continuous belt throughout the New World from northern Mexico southwards into northern South America. Both structural and functional differences differentiate these forests from tropical rainforests [26]. The amount of annual rainfall is a primary distinction between these forests, with TDF experiencing up to eight months of arid-like conditions followed by four months of deluge. Although evidence suggests that TDF may be far more diverse than currently realized [27], habitat fragmentation due to both natural and anthropogenic factors is threatening the evolutionary potential of species inhabiting this ecosystem [28], [29]. Habitat fragmentation is of particular concern in rapidly developing countries such as Mexico, where dense continuous forest is being cleared for both livestock and agriculture [28]. In Mexico, TDF reaches its northern limit near Alamos, Sonora, whereas forest density is highest in the southwestern states of Jalisco, Colima, Michoacan, and Guerrero [30]. Fragmentation of these forests is documented to have occurred for decades and continues to increase [31], [32]. Unfortunately, we know relatively little about how fragmentation and other anthropogenic influences affect species and populations distributed throughout these ecosystems.

The diversity of Mexico's herpetofauna is substantial, with approximately 1,000 described species and many more awaiting formal description [27], [33]. Flanking the Pacific Coast, Mexico's TDF also appears to be a centre of endemism for a variety of amphibian and reptilian taxa [34] including many species of leaf-toed geckos of the genus *Phyllodactylus* [35]. These lizards inhabit arid to semi-arid areas from southern California southwards through Middle America into northern South America and into the West Indies. Like many geckos, they are commonly found on vertical surfaces including bridges and buildings. They also appear to be common in close proximity to small streams, suggesting that riparian connectivity may be an important predictor of dispersal patterns.

Herein we use the Mexican yellow-bellied gecko, *Phyllodactylus tuberculosus*, to understand the effects of landscape configuration and anthropogenic influence on functional connectivity for a small terrestrial vertebrate presumably dependent on TDF. This species is an ideal choice to examine the relationship between landscape and genetics for several reasons. First, the geographic distribution of the gecko mirrors the distribution of TDF in Mexico. Second, abundance is relatively high when populations are isolated, providing a statistically suitable model. Third, along with others in the genus, this species is at risk of local and area-wide extirpation due to habitat fragmentation and recent introductions of non-native, all female species, such as geckos of the genus *Hemidactylus*, which appears to be displacing leaf-toed geckos (pers. obs.). Further, local people actively kill leaf-toed geckos as they are presumed to be venomous and dangerous to humans. For these reasons, leaf-toed geckos may soon pose a conservation concern and identifying landscape components that maximize genetic connectivity may be necessary for managing the persistence of populations. Our study site lay in the northernmost limit of TDF near Alamos, Sonora. We specifically test the following hypotheses: 1) high levels of genetic diversity and differentiation occur over small spatial scales; 2) anthropogenic fragmentation influences functional connectivity; 3) riparian connectivity predicts dispersal patterns, yet 4) some rivers act as dispersal barriers. We also test for an influence of slope and temperature on genetic patterns because the species is generally restricted to warm lowland habitats. Testing these hypotheses and drawing robust conclusions requires principles and practices drawn from diverse research disciplines [16]. Therefore, we use recent advances in landscape genetic techniques [18], [36], [37] to test our hypotheses [38] and identify which landscape variables influence functional connectivity in leaf-toed geckos. We are particularly interested in adopting multivariate approaches to model selection to assess the relative influence of multiple variables simultaneously [37].

## Materials and Methods

### Sampling

**Figure 1:**
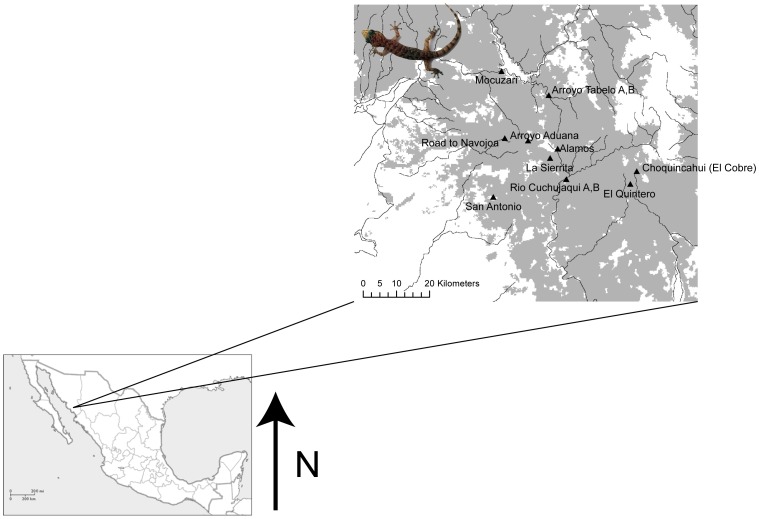
Sampling sites for all individuals and populations of *Phyllodactylus tuberculosus* included in this study. Darker shades of gray represent tropical dry forest. Dark lines represent rivers, streams and arroyos sampled throughout the study area.

Our study area (Fig. 1) has a relatively high degree of forest cover compared to other locations throughout western Mexico due, in part, to the federal protection of land (Sierra de Alamos/Rio Cuchujaqui Reserve). The landscape at lower elevations (generally under 300 m) consists of tropical dry thornscrub that gradually transitions into TDF closer to the Sierra de Alamos with increasing elevation. From 2008 to 2010, we sampled 336 leaf-toed geckos from 12 different localities (mean = 28 individuals per locality) throughout the landscape surrounding the Alamos region (Table 1). Sampling localities were chosen based on landscape characteristics to allow for testing our hypotheses. We sampled on opposite banks of two relatively large rivers or arroyos (Rio Cuchujaqui and Arroyo Tabelo) to test the hypothesis that rivers served as barriers to gene flow (in addition to conduits through opposite banks). Following Animal Use Protocols approved by the Royal Ontario Museum Animal Care Committee, tail tips were taken in the field and immediately preserved in 95% ethanol for subsequent genetic analysis. Subsequently, all individuals were released at the precise site of capture.

**Table 1:** Characterization of genetic diversity of *Phyllodactylus tuberculosus* at each sampling site included in this study based on data from 10 microsatellite loci.

Population	Elevation (m)	n	n(msat)	H_O_	H_E_	# alleles	allelic richness
Road to Navojoa	455	10	10	0.717	0.755	65	65.000
Alamos	375	36	35.2	0.762	0.773	88	64.887
Tabelo A	167	27	26.3	0.701	0.714	70	56.555
Tabelo B	199	30	29	0.721	0.734	69	54.234
Aduana	497	17	16.5	0.756	0.780	75	65.606
Rio Cuchujaqui A	358	30	28.9	0.749	0.762	86	65.451
Rio Cuchujaqui B	261	42	41.3	0.772	0.781	83	64.530
Sierrita	483	38	36.4	0.698	0.707	89	63.881
Mocuzari	124	30	29.6	0.701	0.713	70	55.220
El Quintero	361	30	29	0.690	0.703	83	61.761
Choquincahui (El Cobre)	433	31	29.2	0.709	0.721	81	59.340
San Antonio	388	15	13.6	0.724	0.752	69	62.731

n  =  number of individuals, n(msat)  =  number of individuals accounting for missing data, H_O_  =  observed heterozygosity, H_E_  =  expected heterozygosity; allelic richness calculated based on population with smallest sample size (n = 10).

### DNA extraction and genotyping

DNA was extracted using standard phenol-chloroform procedures. We used polymerase chain reaction (PCR) to amplify 12 polymorphic microsatellite loci developed specifically for *P. tuberculosus* [39]. Both negative and positive controls were run on each PCR plate. PCR products were visualized on an ABI 3730 automated sequencer (Applied Biosystems Inc.) at the Royal Ontario Museum. Genotyping was performed using GeneMarker v.1.95 (SoftGenetics). We re-ran PCRs for approximately 10% of our samples to quantify any potential errors in genotyping.

### Genetic diversity

We calculated diversity statistics for each site including number of alleles and allelic richness using microsatellite analyser v.4.05 [40]. Observed (*H*_O_) and expected (*H*_E_) heterozygosites were calculated in tfpga v.1.3 [41]. We tested for site-specific deviations from Hardy-Weinberg (exact test) and linkage equilibrium using GenePop v.4.0.10 [42], [43]. Significance for tests was assessed using the Markov chain method using 100 batches with 1,000 iterations per batch. We used the false-discovery rate method to control for multiple comparisons [44].

### Genetic differentiation

We calculated both traditional *F*_ST_ and Jost's *D* between populations. Unlike *F*_ST_, *D* measured the degree of allelic differentiation between populations and was particularly useful for highly polymorphic markers such as microsatellites [45]. Microsatellite analyser was used to calculate both global and pairwise multilocus *F*_ST_ [46]. Significance of comparisons was assessed using 10,000 permutations while implementing a Bonferroni correction for multiple tests. We used smogd v.1.2.5 [47] to calculate pairwise *D*_est_ based on harmonic means estimated over all loci. Unbiased estimates of all metrics were employed to account for artefacts of sample size [45], [48].

### Population structure

We tested for the presence of genetic clusters using structure v.2.3.3 [49], [50] to infer population structure. We employed the admixture model with correlated allele frequencies while specifying a range of *K*-values (1–12). For simplicity, we created a batch run specifying the range of *K*-values to be evaluated and implemented 10 independent runs per *K.* Each run was composed of a burn-in of 30,000 followed by 100,000 iterations, which was sufficient to reach convergence. We evaluated the most likely number of clusters using both the ln Pr (*X*/*K*) and the Δ*K* method [51]. Structure harvester [52] was used to visualize outputs and calculate *K* based on both methods. To deal with the multimodality of utilizing multiple independent runs, we used clumpp v.1.1.2 [53] to permute the admixture coefficients for the runs with the chosen *K*-value using the “Greedy” algorithm with 1,000 random input orders. Distruct v.1.1 [54] was then used to visualize the output from clumpp.

Geographic (spatial) information has often provided valuable insights in population genetic structure [55]. Thus, we compared our aspatial structure results to those that incorporated information about sampling locations as prior information [56]. We introduced an additional parameter (LOCPRIOR) into the clustering analysis by specifying a different integer for each sampling location. We then ran structure under the same conditions as the aspatial model.

### Landscape genetic analysis—least-cost paths

We first calculated effective distances between populations using least-cost path modeling [19], [21], [57], [58]. This assessed the influence of different landscape and environmental variables on population genetic structure assuming a single optimal dispersal path. These effective distances were created in a GIS environment by parameterizing different resistance surfaces that represented the hypothesized relationship between a specific habitat feature and gene flow [8]. For example, if hypothesizing that urban development versus undisturbed habitat constrained the movement of individuals, we assigned a higher cost value to cells representing urban habitat.

We tested for the relative influence of several landscape variables on genetic differentiation based on pairwise *D*_est_. Landscape variables were selected based on expert knowledge of which habitat characteristics were most likely important in shaping patterns of gene flow in the species [8]. Our first model was based on isolation-by-distance (IBD) [59], which assumed genetic differentiation was a by-product of simple Euclidean distance without regard to the landscape. Next, we tested a variety of landscape genetic hypotheses that explicitly considered the intervening matrix [10]. First, we tested for the influence of land cover-type (specifically TDF vegetation) on genetic connectivity. We utilized a raster data set produced by the North American Land Change Monitoring System (NALCMS). Nineteen different land cover-types were classified at a 250 m spatial resolution. We created resistance surfaces by reclassifying the data to assign higher cost values to non-forested versus forested habitat. We tested several different cost ratios (1∶2, 1∶10, 1∶100, 1∶1000) to determine how parameterization might have influenced the results. We used the Mantel test function in the R package ecodist [60], [61] to test for both the presence of IBD and to select among the four relative cost values chosen for parameterization. Optimal values were selected based on the Mantel *r* correlation statistic using 10,000 randomizations.

Because *P. tuberculosus* occurred in lowland tropical environments only, our second set of analyses developed least-cost paths based on slope. These data were derived from a GTOPO30 digital elevation model (DEM) with a 1 km^2^ spatial resolution produced from Natural Resources Canada and the U.S. Geological Survey. This layer consisted of seven elevation classes that were reclassified into slope to test the prediction that gene flow occurred primarily throughout lowland habitats. Our study area encompassed an elevational range between 100 to 500 m above sea level. Because slope represented continuous data and because we assumed a linear relationship between slope and gene flow [62], we simply reclassified the data into 32 classes using floating point (i.e. continuous) cell values. Higher cost values were assigned to cells with higher slope. This enabled us to test the prediction that higher slope resulted in lower levels of gene flow or higher genetic differentiation.

Because we often captured geckos adjacent to streams and arroyos, we tested if dispersal occurred primarily via stream corridors. We first obtained a polyline file representing all of Mexico's streams and tributaries from the GISDataDepot, a site that compiled multiple data layers based on ESRI's Digital Chart of the World (DCW). To represent riparian corridors, we created 1 km buffers around stream networks in the polyline file. We then converted these data into a raster file with a cell size of 100 m^2^ and assigned different cost values to cells encompassing buffered streams versus those that did not. We tested the same relative cost values as our land cover analysis (1∶2, 1∶10, 1∶100, 1∶1000) and selected the best values based on Mantel correlations.

We tested for effects of minimum temperature of the coldest period of the year because these lizards are predominantly found in warm tropical lowland habitats. A significant correlation between gene flow and minimum temperatures was predicted. Temperature data were obtained from the WorldClim database at a resolution of 1 km^2^. As with slope, we assumed a linear relationship between temperature and gene flow and we reclassified the data into a continuous distribution with 32 classes. We assigned higher cost values to cells representing lower minimum temperatures.

Finally, we utilized a multivariate resistance surface representing the combined effects of anthropogenic land-use (anthropogenic model). Data were obtained from the Wildlife Conservation Society (WCS) and the Center for International Earth Science Information Network (CIESIN). These data represented the combined effects of population density, built-up areas, roads, railroads, navigable rivers, coastlines, land-use, and nighttime lights (The Last of the Wild, Version Two 2005). The data were categorized based on the Human Influence Index at a spatial resolution of 817 m^2^. Cell values ranged between 0 and 64, with 0 representing no anthropogenic influence and 64 representing maximum influence. To create least-cost paths, we reclassified the data into a continuous distribution with 32 classes and assigned cost values ranging from 0 to 31, with 31 representing the highest cost to gene flow for cells with the highest anthropogenic influence. Like slope and temperature, we assumed a linear relationship between the degree of disturbance and gene flow.

For all least-cost path-analyses, we used the landscape genetics toolbox 1.2.3 [63] implemented in ArcMap 10 to calculate effective distances between sampling localities. This calculated both the cumulative cost-distance and the length of the least-cost path between any two sampling points. Because both distances could have been sensitive to relative cost values [64], we tested several different relative values for categorical variables as described above. For all least-cost path-analyses, we used the cumulative cost-distance because this metric minimized the degree of multicollinearity in our predictors.

### Landscape genetic analysis—circuit theory

We also modelled patterns of gene flow using circuit theory [65]–[67]. This so-called isolation-by-resistance (IBR) approach has been shown to be powerful in modeling functional connectivity in both simulated and empirical data sets [65], [66]. We calculated resistance distances between populations using circuitscape 3.5.7 [68]. Each calculation used focal points in pairwise mode and an eight-neighbors connection scheme. Due to memory issues with the original 100 m stream data, we aggregated cells in this raster to a resolution of 200 m to obtain reasonable computing times. Resistance distances based on all other variables were calculated using the original resolution of the data layer (e.g. 250 m for the land cover). All calculations were based on values of per-cell resistance.

### Statistical analysis

We used multiple regression analyses on distance matrices (MRM; [69], [70]) in ecodist to evaluate landscape-genetic relationships. Although a potentially powerful method for landscape-genetic inference [37], few studies have incorporated MRM analyses (e.g. [71]). Similar to the commonly used partial Mantel test [72], MRM was developed to test for significant relationships between a dependent distance matrix (e.g. linearized *D*_est_) and a number of indicator matrices and identify the contribution of each explanatory variable to the overall fit of the model [69]. Further, MRM modeled polynomial and nonlinear relationships [70]. Each distance matrix was unfolded into vectors representing pairwise distances. The response vector (i.e. linearized *D*_est_) was then regressed against each indicator vector (i.e. least-cost or resistance distances) and the significance of the model was assessed by permuting the objects of the response vector.

MRM models using all six explanatory variables were not created for the least-cost analyses due to a relatively high degree of collinearity among the predictors, which could have resulted in coefficients with large variances and lead to erroneous conclusions regarding the direction and magnitude of slope. Some authors have suggested calculating Variance Inflation Factors (VIF) for each predictor in a model to ascertain if collinearity might be a problem in parameter estimation [73]. Like previous studies, we used VIF values>10 as evidence for substantial multicollinearity [71]. Thus, for least-cost paths we selected candidate models (see below) based on both our hypotheses of interests and to minimize the potential error in estimated regression coefficients. Because multicollinearity was minimal with our resistance distances calculated from circuitscape, we included all variables in the model selection procedure. We predicted a negative relationship between genetic differentiation and several of our landscape features including stream networks and the degree of undisturbed habitat. Because multiple regression models account for the effects of all included predictors, we anticipated that the regression coefficients may change depending upon the other variables included in the model (e.g. Euclidean distance). Univariate MRM models all resulted in positive coefficients due to spatial autocorrelation. Thus, particular attention was placed on regression coefficients in highly supported models containing Euclidean distance as a predictor.

We utilized information theoretic criteria to select among candidate models hypothesized to be important predictors of spatial genetic variation in our system [74]. Specifically, we calculated second-order AIC values (AICc) for competing candidate models based on either least-cost or resistance distances. Candidate models were selected based on a priori hypotheses regarding which combination of variables best explained patterns of genetic structure. In all cases we tested fewer than 20 candidate models [75]. The best model minimized the amount of information lost as represented by the combination of variables with the lowest AICc value [76]. Different combinations of variables were compared to the null model of IBD to determine if the incorporation of landscape variables explained more of the variation in *D*_est_. We followed previous recommendations in assessing the relative importance of models [75]. We also used MuMIn [77] to calculate AICc weights for each model and we estimated the 95% confidence set of candidate models [74].

Because it remained unknown how information theoretic criteria performed when evaluating models based on pairwise distances, we compared our MRM results with a linear mixed modeling approach [78]. This approach was based on a maximum likelihood population effects (MLPE) model that explicitly accounts for non-independence of values in regressions on distance matrices [79]. We created linear mixed models using the R package lme4 [80] defining populations as the random effect and each predictor matrix as a fixed effect. Parameter estimation was performed using restricted maximum likelihood (REML). All predictor matrices were centered around their mean prior to analysis. Statistical significance of both fixed and random effects were calculated using the R package MixMod [81]. Finally, to select among competing models, we calculated R^2^_β_ [82] for each model based on the Kenward-Roger F and degrees of freedom [83] calculated using the R package pbkrtest [84]. MLPE models were calculated for the top set of candidate models as determined from the MRM analysis to compare relative performance.

## Results

### Genetic diversity

All microsatellite data were deposited in Dryad (Provisional DOI: doi:10.5061/dryad.tj1k5). The genotyping and scoring of microsatellite alleles had an error rate of less than 1%, and, thus, high reproducibility. After controlling for false discovery rates, some loci showed significant deviations from Hardy-Weinberg expectations within collecting sites. For example, locus G2_96 showed heterozygote deficits at six of the 12 sites, locus P7 at eight of 12, and locus G2_59 at four of 12 (Table S1). However, only three alleles were present at locus G2_59 and, thus, there was a high probability that random chance resulted in significance. Because loci G2_96 and P7 showed a significant heterozygote deficit at multiple sites, we ran preliminary analyses with and without these loci to see how results changed. Although results did not differ substantially, we adopted a conservative approach and chose to report results from subsequent analyses excluding these two loci.

After controlling for false discovery rates, a few loci showed signs of linkage disequilibrium. For example, locus G2_22 showed linkage to loci G2_96, P2, P7, P12, and P19. Locus G2_85 showed signs of linkage with locus P7, and locus P2 with P15. However, linkage occurred at only two of our 12 sites (Arroyo Tabelo B and Mocuzari) suggesting that our loci were, in fact, independent, unlinked markers.

**Figure 2:**
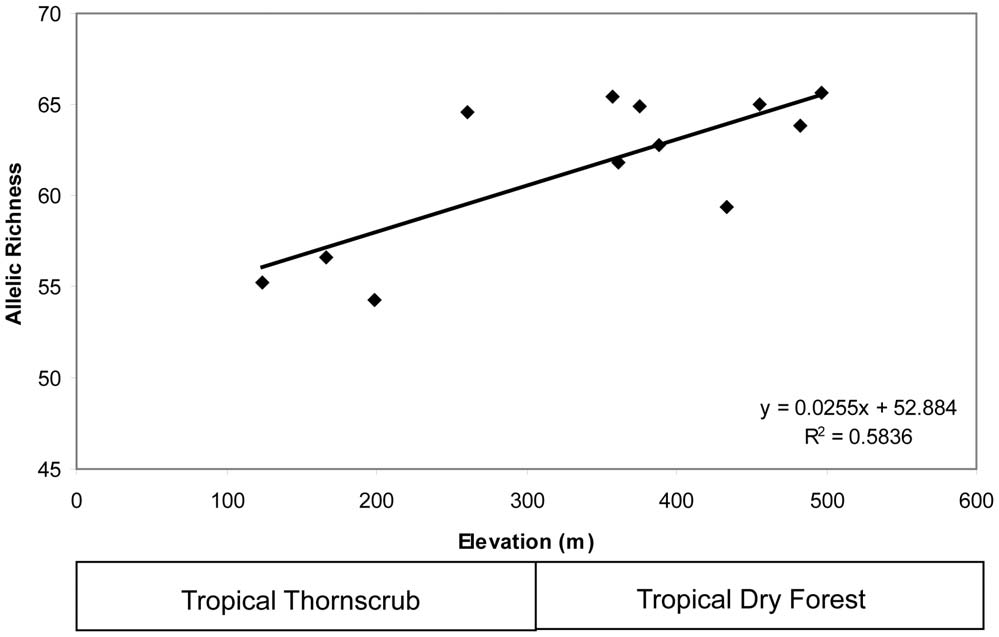
Relationship between elevation and genetic diversity (allelic richness) for all populations of *Phyllodactylus tuberculosus* included in this study.

In general, within-site diversity was moderate as shown by both expected heterozygosity and allelic richness (Table 1). Expected heterozygosity ranged from 0.7025 to 0.7812 and allelic richness from 54.2341 to 65.6058. Allelic diversity within loci over all populations ranged from three alleles at locus G2_59 to 26 alleles at locus G2_37 (mean number of alleles per locus = 13.1). In general, diversity estimates were fairly similar among sites. A highly significant positive relationship occurred between elevation and genetic diversity that generally corresponded to habitat-type (tropical thornscrub versus TDF; R^2^ = 0.5836;P = 0.004; Fig. 2).

### Genetic differentiation

Moderate levels of global genetic differentiation were resolved based on both *F*_ST_ (F_ST_ = 0.0869;P = 0.0001) and *D* (*D*_est_ = 0.248). Pairwise measures of differentiation revealed moderate to high levels of population divergence (Table 2). Pairwise *F*_ST_ values ranged from zero between Aduana and the road to Navojoa (RN) to 0.201 between La Sierrita and El Quintero. Further, the majority of *F*_ST_ values were significant based on 10,000 random permutations of alleles after a Bonferroni correction (P<0.00075). Values of *D*_est_ ranged from moderate to high, showing similar relative values between populations. El Quintero and Choquincahui were the most divergent from the remaining populations based on both statistics. We found a high correlation between *F*_ST_ and *D*_est_ (Pearson r = 0.940). Because of this correlation, all subsequent landscape genetic analyses were performed with *D*_est_ only. We detected a significant positive correlation between Euclidean distance and *D*_est_ (Mantel r = 0.4897, P<0.001).

**Table 2:** Pairwise genetic differentiation between populations estimated from 10 microsatellite loci.

	Road to Navojoa	Alamos	Tabelo A	Tabelo B	Aduana	Cuch A	Cuch B	La Sierrita	Mocuzari	El Quintero	Choquincahui	San Antonio
Rd to Navojoa	--	0.106	0.181	0.174	0.000	0.119	0.075	0.048	0.094	0.325	0.311	0.113
Alamos	0.032	--	0.197	0.166	0.072	0.086	0.095	0.139	0.151	0.217	0.309	0.032
Tabelo A	**0.080**	**0.080**	--	0.050	0.125	0.196	0.223	0.257	0.133	0.260	0.309	0.248
Tabelo B	**0.065**	**0.059**	0.026	--	0.143	0.224	0.232	0.252	0.100	0.293	0.377	0.197
Aduana	−0.003	0.021	**0.058**	**0.059**	--	0.074	0.084	0.069	0.123	0.207	0.269	0.083
Cuch A	**0.039**	**0.028**	**0.082**	**0.076**	**0.024**	--	0.059	0.131	0.150	0.170	0.195	0.134
Cuch B	**0.033**	**0.029**	**0.087**	**0.075**	**0.031**	**0.021**	--	0.137	0.197	0.236	0.319	0.101
La Sierrita	**0.042**	**0.056**	**0.154**	**0.123**	**0.047**	**0.078**	**0.069**	--	0.176	0.345	0.378	0.155
Mocuzari	**0.045**	**0.063**	**0.068**	**0.049**	**0.056**	**0.055**	**0.070**	**0.097**	--	0.267	0.318	0.172
El Quintero	**0.149**	**0.118**	**0.147**	**0.151**	**0.128**	**0.096**	**0.111**	**0.201**	**0.150**	--	0.009	0.217
Choquincahui	**0.140**	**0.122**	**0.142**	**0.150**	**0.125**	**0.092**	**0.114**	**0.199**	**0.142**	0.008	--	0.336
San Antonio	0.040	0.019	**0.099**	**0.078**	0.027	**0.040**	**0.042**	**0.069**	**0.083**	**0.135**	**0.144**	--

Values above diagonal represent *D*_est_ and values below diagonal *F*_ST_. Bold values of *F*_ST_ indicate significance (P<0.00075) after Bonferroni correction.

### Population structure

**Figure 3:**
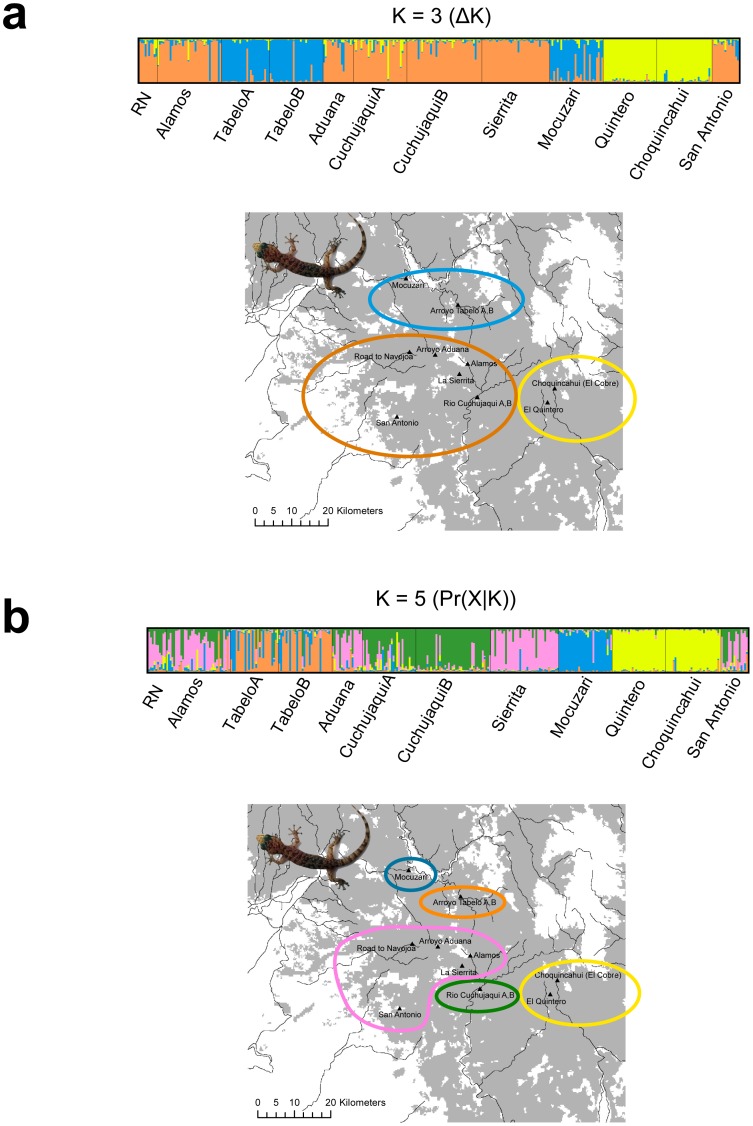
Structure results excluding prior information on sampling locality for *Phyllodactylus tuberculosus* included in this study. a) Results for *K* = 3 selected using the Δ*K* method. b) Results for *K* = 5 selected using ln Pr(*X|K*). Each vertical bar represents a single individual with different colors representing the proportion of an individual's genome originating from that specific cluster. Names below plots represent population codes referred to in the text. Colored circles represent the most likely ancestry.

An optimal *K*-value based on Δ*K* suggested a *K* = 3 (Δ*K* = 138.332; Fig. 3a; Fig. S1b). A *K*-value of 5 was obtained using the ln Pr(*X*|*K*) method (Fig. 3b; Fig. S1a). Individuals on opposite banks of Rio Cuchujaqui and Arroyo Tabelo did not form distinct clusters. However, individual-based Mantel tests found a highly significant barrier effect for both Rio Cuchujaqui (Mantel r = 0.1148; P <0.0001) and Arroyo Tabelo (Mantel r = 0.1126; P = 0.0006).

**Figure 4:**
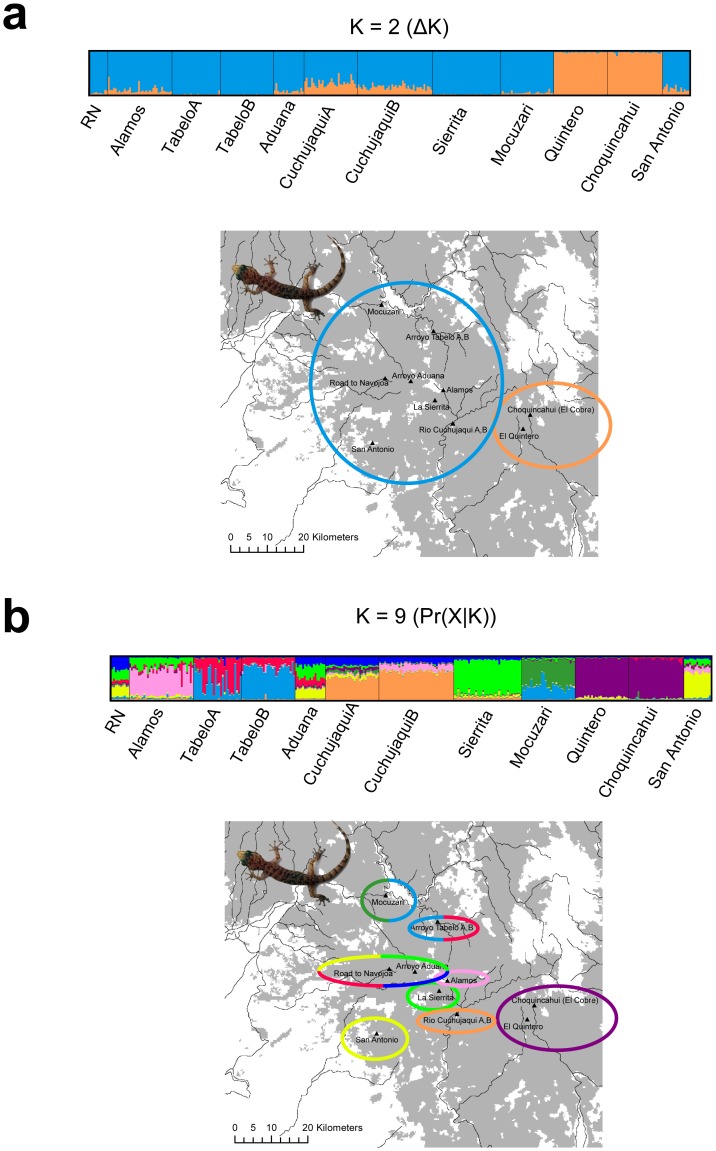
Structure results including prior information on sampling locality for *Phyllodactylus tuberculosus* included in this study. a) Results for *K* = 2 selected using the Δ*K* method. b) Results for *K* = 9 selected using ln Pr(*X|K*). Each vertical bar represents a single individual with different colors representing the proportion of an individual's genome originating from that specific cluster. Names below plots represent population codes referred to in the text. Colored circles represent the most likely ancestry.

A second set of analyses utilized a model that explicitly incorporated prior information for sampling localities to aid in clustering. These results differed from the clustering results that did not utilize sampling localities. For example, whereas the Δ*K* method suggested a *K* of 2 (Δ*K* = 80.915; Fig. 4a; Fig. S2b), the plot of *K* versus ln Pr(*X*|*K*) reached a slight peak at 9 (Fig. 4b; Fig. S2a). For *K* = 9, some structure was resolved across opposite banks of Rio Cuchujaqui and Tabelo (Fig. 4b). The plot of *K* versus ln Pr(*X*|*K*) showed that likelihood values began to stabilize at about *K* = 3, which was a value similar to that chosen using the Δ*K* method in the aspatial analysis. At *K* = 3, cluster memberships and admixture coefficients were very similar to those of the aspatial analysis.

### Landscape genetics—least-cost paths

For both forest cover and streams, Mantel *r* values were highest with a cost ratio of 1∶2 (respectively Mantel r = 0.4344, P = 0.002; r = 0.6090, P<0.001). The MRM analysis based on least-cost path distances suggested that the incorporation of landscape variables explained significantly more variance in genetic differentiation than a simple IBD model (Table 3; Table S2). Whereas Euclidean distance was able to explain approximately 44% of the variation in *D*_est_ values, the incorporation of landscape variables increased this value to approximately 62%. The statistically best supported model (using AICc weights) was based on a combination of Euclidean distance, stream connectivity, and the degree of anthropogenic disturbance (R^2^ = 0.621; w_i_ = 0.856). Our least-cost paths based on temperature and slope explained slightly more variation in *D*_est_ than Euclidean distance (0.548 and 0.539 versus 0.44, respectively; Table S1). Least-cost paths based on forest connectivity received relatively little support with similar weights to the Euclidean distance model. However, these two distances were highly correlated (Mantel r = 0.995; P<0.001) leading to very large VIF values. After accounting for Euclidean distance in models, regression coefficients for stream connectivity, forest, and undisturbed habitat (anthropogenic model) were negative and, thus, associated with a lower *D*_est_ and higher gene flow.

**Table 3:** Multiple regression on distance matrices (MRM) and maximum likelihood population effects (MLPE) results showing the relationship between pairwise genetic distance (linearized *D*_est_) and least-cost path cost distances incorporating landscape heterogeneity.

Model	Variables	β	P	Model R^2^	P	VIF	Model AICc	ΔAICc	Akaike Weight (*w_i_*)	β (MLPE)	P (MLPE)	R^2^_β_	P_RE_
A	Euclidean	2.75E-05	0.0014	0.621	0.0001	**20.26**	−118.13	0.00	0.856	1.89E-05	<2E-16	0.517	0.0200
	Anthropogenic	−1.72E-06	0.0077			4.20				−1.12E-06	<2E-16		
	Stream	−6.77E-06	0.1152			**13.64**				−4.11E-06	0.0090		
B	Euclidean	1.62E-05	0.0002	0.573	0.0001	3.86	−112.60	5.53	0.054	1.16E-05	<2E-16	0.467	0.0100
	Anthropogenic	−1.46E-06	0.0426			3.86				−8.42E-07	<2E-16		
C	Temperature	1.99E-06	0.0052	0.566	0.0001	5.39	−111.53	6.60	0.032	1.09E-06	<2E-16	0.454	0.0020
	Stream	−2.65E-06	0.3308			5.39				2.01E-07	0.0300		
D	Temperature	1.44E-06	0.0001	0.548	0.0001		−111.06	7.07	0.025	1.14E-06	<2E-16	0.446	0.0050

Candidate models tested were based on a priori hypotheses and to minimize collinearity among predictors. For clarity, only models with relatively high support based on ΔAICc and *w_i_* are shown (i.e. confidence set of candidate models; [74]). Optimal cost values used to parameterize resistance surfaces prior to calculating each least-cost path were selected based on Mantel *r* correlation coefficients. VIF = Variance Inflation Factor. P_RE_ represents P-value for population effect.

Similar results were obtained from the MLPE models with a model containing Euclidean distance, anthropogenic disturbance, and stream connectivity receiving the highest support (R^2^_β_ = 0.517; Table 3). All fixed effects for each model were statistically significant as was the population (random) effect. In general, the sign of regression coefficients was identical between MRM and MLPE models. However, for Model C stream had a negative coefficient for MRM and a positive coefficient in MLPE. The relative support for top models was the same for both MRM and MLPE.

### Landscape genetics—circuit theory

**Figure 5:**
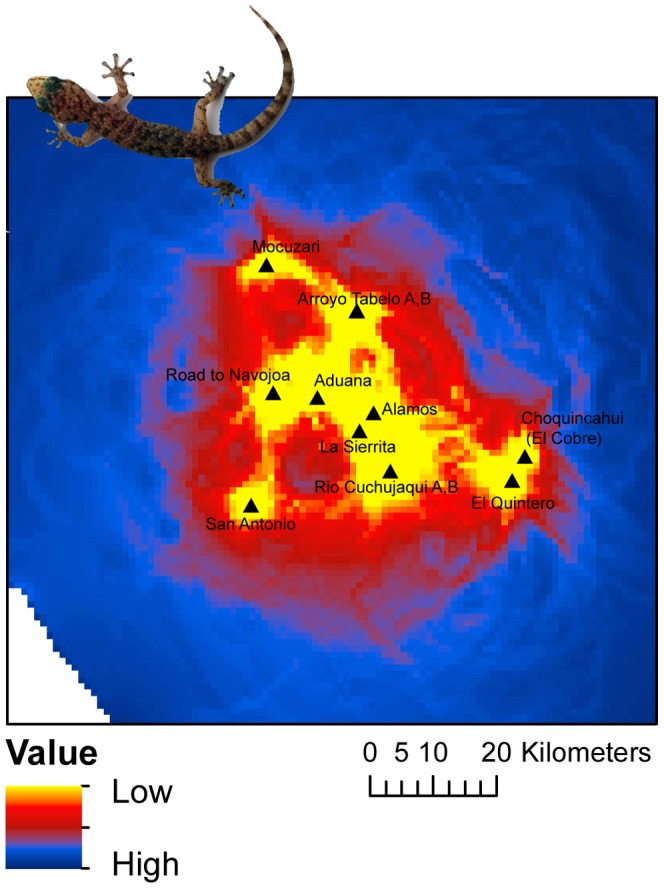
Results from circuitscape depicting cumulative resistance between populations of *Phyllodactylus tuberculosus* based on the combined effect of Euclidean distance, slope and riparian connectivity. ‘Low’ in figure legend represents low levels of resistance whereas ‘High’ represents high levels of resistance.

Results of MRM based on resistance distances were similar to those based on least-cost path distances (Table 4). However, slope appeared to be an important variable influencing gene flow under a circuit-theoretic approach. The best supported model based on AICc weights included Euclidean distance, slope, and stream connectivity (w_i_ = 0.585), with stream being the only variable with a negative coefficient. The next most supported model, which included all six variables, received considerably less support (ΔAICc = 2.37; w_i_ = 0.167), although well within the estimated confidence set. Visual examination of cumulative current based on a composite map of Euclidean distance, stream networks, and slope was highly congruent with the genetic clusters inferred from the structure analysis (Fig. 5). In most cases AICc weights were higher for multivariate models incorporating landscape variables versus a model of simple IBD (Table S3). VIF values were less than 10 for all models suggesting that multicollinearity among predictors was not likely to be a problem.

**Table 4:** Multiple regression on distance matrices (MRM) and maximum likelihood population effects (MLPE) results showing the relationship between pairwise genetic distance (linearized *D*_est_) and resistance distances incorporating landscape heterogeneity.

Model	Variables	β	P	Model R^2^	P	VIF	Model AICc	ΔAICc	Akaike Weight (*w_i_*)	β (MLPE)	P (MLPE)	R^2^_β_	P_RE_
A	Euclidean	7.81E-06	0.0005	0.626	0.0001	1.65	−118.94	0	0.545	4.95E-06	<2E-16	0.615	3.00E-04
	Slope	9.00E-02	0.0070			1.77				1.19E-01	<2E-16		
	Stream	−1.95E-01	0.0734			2.11				−1.28E-01	0.0010		
B	Euclidean	8.47E-06	0.0006	0.654	0.0003	2.18	−116.57	2.37	0.167	4.75E-06	<2E-16	0.673	9.00E-05
	Anthropogenic	−2.03E-02	0.4354			3.31				7.61E-03	0.1200		
	Stream	−2.02E-01	0.1782			3.33				−8.56E-02	0.0700		
	Forest	8.64E-02	0.7342			4.62				−1.04E-01	0.9700		
	Slope	4.74E-02	0.4369			6.48				1.47E-01	0.0400		
	Temperature	2.75E-02	0.5779			8.30				−1.19E-02	0.3200		
C	Euclidean	7.90E-06	0.0006	0.626	0.0002	1.92	−116.56	2.38	0.166	5.20E-06	<2E-16	0.624	2.00E-04
	Forest	−1.83E-02	0.9152			2.52				−5.99E-02	0.3000		
	Stream	−1.87E-01	0.1851			3.21				−8.95E-02	0.1000		
	Slope	8.92E-02	0.0111			1.88				1.17E-01	<2E-16		
D	Euclidean	8.32E-06	0.0003	0.606	0.0001	2.06	−115.53	3.41	0.099	5.11E-06	<2E-16	0.568	1.00E-05
	Slope	5.05E-02	0.0670			1.47				8.60E-02	<2E-16		
	Anthropogenic	−2.53E-02	0.1106			1.54				−9.21E-03	2.00E-04		

Candidate models tested were based on a priori hypotheses. For clarity, only models with relatively high support based on ΔAICc and *w_i_* are shown (i.e. confidence set of candidate models; [74]). Optimal cost values used to parameterize resistance surfaces prior to calculating resistance distances were selected based on Mantel *r* correlation coefficients. VIF = Variance Inflation Factor. P_RE_ represents P-value for population effect.

Relative support for top candidate models differed between MRM and MLPE. Unlike MRM, MLPE favored the model containing all predictors (R_β_^2^ = 0.673) versus a model with Euclidean distance, slope, and stream connectivity (R^2^_β_ = 0.615; Table 4). However, in the former many of the fixed effects were not significant. In general, direction of slope was similar for both MRM and MLPE models. Incongruence was only detected in models with non-significant predictors. The population (random) effect was highly significant in all models.

## Discussion

This study highlights the power of adopting a landscape genetics approach to understanding functional connectivity for tropical organisms. We show how the incorporation of landscape heterogeneity into our models can enhance our understanding of processes shaping the genetic structure of populations. Rates of deforestation and habitat fragmentation are increasing exponentially throughout these regions. Coupled with climate change, we still know relatively little about how organisms will respond to these continuing threats [32], [85]–[87]. Based on our results we cannot reject the hypothesis that landscape composition is an important predictor of spatial genetic variation in this system. This suggests that if we are to fully comprehend how natural and anthropogenic habitat alteration influences functional connectivity in tropical organisms, additional landscape genetic studies are required to infer species-specific responses to continued habitat change.

### Genetic diversity and population structure

Our results detect moderate to high levels of genetic diversity within populations of *P. tuberculosus*. Diversity is not substantially higher in the population near Alamos versus the other areas, as might be predicted given the tendency for many gecko species to aggregate near human settlements. Further, a statistically significant relationship occurs between elevation and allelic richness. These results corroborate field observations that these lizards are predominantly encountered in TDF habitat. For example, elevation of our sites ranges from 100 m to 500 m. This range in elevation spans two distinct habitat types: tropical thornscrub and TDF [27]. An abrupt change in tropical vegetation occurs at approximately 400 m as well as an apparent change in abundance of geckos; more individuals are encountered per unit of time at El Quintero (361 m) and Choquincahui (433 m) than the other sites.

Significant population structure occurs based on both pairwise differentiation statistics and results from structure. Most pairwise *F*_st_ values are high and statistically significant, with the localities El Quintero and Choquincahui being most divergent from all other populations. Values of *D*_est_ yield similar results. Thus, leaf-toed geckos appear to exhibit substantial population differentiation over relatively fine spatial scales.

Considerable recent debate exists as to the best *F*_ST_-like analogue for assessing genetic differentiation between populations [48], [88]–[90]. The traditional *F*_ST_ metric (*G*_ST_ for multiple loci and alleles) is highly sensitive to within-population heterozygosity, making it difficult to compare values between studies and markers [45], [91]. Further, because *F*_ST_ is dependent on the number of alleles and heterozygosity, two populations can have low *F*_ST_ values despite sharing no alleles. For example, studies have shown that in two hypothetical populations with a total of 16 alleles, *F*_ST_ is constrained to less than 0.1 even when the two populations share no alleles [90]. New metrics such as *G*'_st_ [91] and *D* [45] circumvent some of the more common issues found with *F*_ST_ [92]. Although these new metrics are not without criticism [93], a comparison of multiple statistics can maximize information [92]. Whereas *D* is a useful metric for landscape-genetic inference, spatially explicit computer simulations may provide a greater understanding of how this measure compares to other commonly used differentiation statistics.

Our structure analyses reveal cryptic population structure. We infer values of *K* both with and without incorporating prior information on sampling localities. Incorporating locality data as a prior changes our inference of *K*. The Δ*K* method suggests *K* = 2, whereas the plot of *K* versus ln Pr(*X*|*K*) suggests *K* = 9. Populations at El Quintero and Choquincahui always group together into a single cluster and the populations at Mocuzari and Tabelo fall into separate clusters from the remaining populations in several Structure analyses. Structure plots generally corroborate the levels of differentiation based on *D*_est_ and *F*_ST_.

We never detect structure on opposite banks of the Río Cuchujaqui or Arroyo Tabelo based on aspatial Bayesian clustering (i.e. individuals on opposite banks cluster as one group with similar admixture coefficients). However, spatial clustering at *K* = 9 reveals different ancestries on opposite banks. Further, Mantel tests suggest a significant barrier effect for these features. Although Bayesian clustering methods, and structure in particular, can be a powerful tool for inferring recent linear barriers to gene flow [94], [95], their high Type-1 error rates, relatively low power, and difficulty in interpretation require caution when using these methods to test barrier hypotheses. Of interest is that we were able to detect a barrier effect even though on several occasions we witnessed geckos dispersing across the underside of bridges. This suggests that some individuals are crossing streams and suggests that Mantel tests may be the most sensitive to detect a relatively weak barrier effect.

New, sophisticated algorithms achieve difficult genetic clustering. Although early algorithms are entirely aspatial in nature [49], recent applications incorporate prior locality information into the analysis [96]–[99]. Structure incorporates geographic information by assigning different codes to different populations [56]. Although relatively underutilized, this method is appealing in cases like ours where geographic information is available for populations and not individuals. Different Bayesian clustering programs often obtain different results [94], [95] and this necessitates additional empirical and simulation studies to test the power of the spatial approach in structure in comparison to the fully spatial models implemented in other software packages.

To date, few studies examine the population genetic structure of other lineages of geckos. For example, a recent study on two species of sympatric gecko species report significant differences in genetic diversity and structure [100]. Their results suggest different dispersal abilities in sympatric species, with one species exhibiting a maximum dispersal distance of only 500 m. The results of our analysis of *P. tuberculosus* also suggest that many gecko species may have limited dispersal abilities and rely on landscape characteristics to facilitate dispersal.

### Landscape genetics: least-cost paths

To date, few studies investigate landscape-genetic relationships in Neotropical vertebrates [18], [101]. Our results identify several landscape variables important in shaping the genetic connectivity of leaf-toed geckos. Landscape variables explain significantly more variation in genetic differentiation than IBD. For example, our qualitative observations of relative abundance in different habitats suggest that forest fragmentation will have detrimental effects on functional connectivity. Several landscape genetic studies also report a negative relationship between forest fragmentation and genetic connectivity in small vertebrates [62], [102], but most of these focus on temperate systems. After controlling for Euclidean distance, our least-cost path results show a negative relationship between forest connectivity and genetic differentiation, suggesting that gene flow is higher through forest patches (Table S2). However, we view these results with caution for several reasons. First, models including forest are less well-supported than alternative candidate models. Second, VIF values are exceptionally high for these models and this may be causing large variances in regression coefficients. Third, our study area contains a relatively large amount of undisturbed forest compared to localities in southern Mexico. Thus, Euclidean distances and least-cost path distances based on forest are nearly identical. Additional landscape genetic studies are necessary in areas experiencing rapid loss of TDF in order to understand the effect of forest patch dynamics on functional connectivity.

Our least-cost path analysis suggests that anthropogenic disturbance is influencing functional connectivity in geckos. Because we parameterize the multivariate anthropogenic resistance surface by assigning higher costs to disturbed areas, the significantly negative regression coefficient (after controlling for Euclidean distance) suggests that undisturbed habitat is associated with a lower *D*_est_ or higher gene flow. These results are concordant with other studies that show a negative relationship between anthropogenic disturbance and rates of gene flow [103]–[105]. This concordance suggests that although geckos are frequently encountered in close proximity to human settlements, these areas have a detrimental impact on genetic connectivity.

Although geckos are common on abandoned houses in TDF habitat, individuals of *P. tuberculosus* are only present in and around houses in absence of introduced geckos of the genus *Hemidactylus* (pers. obs.). Very few leaf-toed geckos can be found syntopically with *Hemidactylus* and on one occasion we witnessed the head of a *P. tuberculosus* in the jaws of *H. frenatus*. Thus, in heavily anthropogenically influenced areas, it appears that introduced *Hemidactylus* are directly competing with native *Phyllodactylus*. To exacerbate this issue, people often kill leaf-toed geckos on site as they believe the darker colour of these geckos indicates that they are venomous. Conservation efforts should focus on educating local people on differences between native and non-native flora and fauna to aid in the maintenance and protection of the native species. This is especially important for species commonly found close to human settlements.

The minimum temperature of the coldest period influences gene flow, which explains slightly more of the variance in *D*_est_ than Euclidean distance (0.548 vs. 0.444). TDF is a seasonal forest with approximately eight months of warm, wet conditions and four months of dry, cooler conditions [26]. These geckos commonly occur in hot tropical lowland environments, and less gene flow will occur in localities that experience lower temperatures. Our MRM and MLPE results suggest that these geckos are avoiding areas experiencing colder temperatures. Seasonality and climate are important variables shaping connectivity for other species [106]–[109]. Future studies will identify if this is a common trend in temperature-dependent species and if/how patterns will change with the continual threat of global warming.

Many geckos are from near bridges and rocky outcroppings adjacent to streams and rivers. Thus, we test the hypothesis that riparian networks are an important component shaping patterns of gene flow. Our results suggest that riparian connectivity is an important predictor for patterns of dispersal, as this variable always occurs in our top models. However, these features may also serve as a genetic barrier. Thus, it appears as if geckos may disperse along riparian networks, but seldom cross them. Previous studies conflict as to the role riparian networks play in shaping functional connectivity in small terrestrial vertebrates. For example, streams facilitate gene flow among populations of blotched tiger salamanders (*Ambystoma tigrinum melanosticum*) [19] as they do in Rocky Mountain tailed frogs, (*Ascaphus montanus*) [110], and Pacific jumping mice, (*Zapus trinotatus*) [20]. Conversely, gene flow occurs terrestrially in coastal tailed frogs (*Ascaphus truei*) and does not follow riparian corridors [62]. Combined, these results illustrate the utility of a GIS-based landscape genetic approach to understanding the influence of stream networks on genetic connectivity of small terrestrial vertebrates and reaffirm the necessity for examining species-specific processes [18]. Nevertheless, evidence suggests that riparian corridors should be given conservation priority in many cases.

### Landscape genetics: circuit theory

Our results based on resistance-distances derived from a circuit theoretic approach are similar to those based on least-cost paths. However, slope becomes a more important predictor of genetic variation in the former models, where populations separated by higher slopes experience lower rates of gene flow (Table 4). These results are similar to numerous other studies that show a direct relationship between topological relief, elevation, and slope on rates and patterns of gene flow in terrestrial vertebrates [19], [111]–[113]. Riparian connectivity and anthropogenic disturbance also appear as important components based on resistance-distances. Similar to the least-cost models, we find relatively weak evidence for an influence of forest structure on genetic connectivity. However, when forest is included in a model with Euclidean distance its coefficient is negative, suggesting that intact forest may facilitate gene flow.

Circuit theoretic approaches complement least-cost path modeling and often explain more of the variance in genetic differentiation than more traditional methods [66], [67]. For example, slope becomes an important predictor of genetic differentiation in our resistance models. This makes sense intuitively and biologically because geckos are unlikely to disperse along one narrow strip of optimal slope. Thus, the application of circuit theoretic approaches is particularly attractive when a single optimal dispersal route is unlikely. The relative utility of least-cost versus resistance distances will likely depend on the scale of the study and the specific landscape feature in question. However, testing for congruence with both approaches will result in more robust conclusions regarding the influence of specific landscape features. Our model with the largest AICc weight (0.626) and R^2^_β_ (0.673) based on resistance distances explains similar variation in *D*_est_ as our least-cost models. However, unlike the least-cost analysis, we can combine all resistance distances into our models due to the lack of collinearity among the predictors. Our results corroborate previous findings and suggest that a combination of circuit theoretic and least-cost models provides a powerful tool for investigating functional connectivity in dynamic landscapes.

### Statistics and landscape genetics

Landscape genetics is still a relatively new discipline [16] and a large number of recent studies focus on testing the power of various analytical techniques for understanding the influence of landscape variables on microevolutionary processes [37], [38], [94], [95], [114], [115]. Although the Mantel and partial Mantel tests continue to be the most widely used methods to link landscape and genetic data [18], recent research suggests that these methods suffer from low power and high Type-1 errors [37], [116]. Recognizing these limitations, recent studies have concluded that partial Mantel tests implemented in a causal modeling framework are a powerful tool [38], [114]. However, landscape-genetic relationships are often multivariate and are best represented in models that simultaneously consider multiple landscape and environmental variables [8]. Thus, a MRM approach serves as a powerful method to understanding the complex suite of factors important in shaping the spatial distribution of genetic variation [37]. Our study highlights the value of applying MRM analyses to both least-cost and resistance distances. Surprisingly, few studies use this analytical method [71].

Although powerful, MRM approaches have limitations that need to be addressed [69], [70]. Collinearity often occurs among independent variables because they are in the form of distances. Often, this will manifest itself in least-cost analyses and its severity depends on landscape structure and the chosen distance metric [37]. Multicollinearity will not affect predictions of the variance of the dependent variable, but it may have a consequence on individual regression coefficients because of higher standard errors. Regression coefficients for predictors can change drastically depending on what other predictors are included in the model [69]. Researchers implementing MRM should examine the influence of multicollinearity on model results and how regression coefficients change with different models. Rigorous model-selection criteria, such as information theoretic or stepwise regression methods, can identify the best combination of explanatory variables.

Although multicollinearity occurs in our least-cost path data, coefficients change little with different explanatory models (Table 3; Table S2). Thus, we are confident in our conclusions regarding landscape-genetic relationships in this system. In cases where coefficients change drastically between models, VIF values will identify the degree of correlation among the predictors. We recommend that future landscape genetic studies using methods such as MRM and Mantel tests report VIF for each model examined. We also encourage the exploration of ridge regression techniques for landscape-genetic inference.

It remains unclear how the pairwise nature of distance matrices can influence model-selection using information theoretric metrics such as AIC, which generally assume independent observations. Other recent studies acknowledge this potential issue and propose alternatives through the use of Delaunay triangulation [102] or linear mixed models [78]. Unfortunately, the former drastically simplifies landscape heterogeneity while model selection in linear mixed models brings another component of statistical uncertainty. Further, both the sign and magnitude of coefficients may differ depending on the method of analysis used (Table 3,4). The relative performance of these methods for landscape-genetic inference requires evaluation using spatially explicit simulations before strict recommendations can be made.

### Conservation implications

Habitat fragmentation and extirpation continue to threaten tropical ecosystems throughout the globe [86], [117]. Fragmentation of TDF in Mexico is of particular concern as these forests form the predominant vegetation-type and are known to be a biodiversity hotspot [27], [30], [31]. A time-series analysis of Mexican TDF reported that by 1990 only 27% of intact forest remained due to unabated anthropogenic conversion for agriculture and pastureland [28]. The TDF near Alamos constitutes one of the most undisturbed tracts of continuous forest in Mexico due, in part, to federally protected reserves [27]. However, even in areas of relatively high forest cover, slight anthropogenic disturbance may have detrimental impacts to functional connectivity. Our study highlights the need for additional landscape genetic studies focusing on TDF ecosystems to better understand how habitat fragmentation and climatic change will influence ecological and evolutionary processes. To this end, researchers should focus on developing geospatial data sets at finer spatial resolutions. This will allow a far more comprehensive examination of the effect of landscape-level processes on the spatial distribution of genetic variation. Analyses incorporating high-resolution landscape layers, highly polymorphic genetic markers, and sophisticated analytical techniques will allow the design of movement corridors to maximize functional connectivity for species inhabiting this threatened ecosystem.

## Supporting Information

Figure S1 **a) Structure results illustrating changes in ln Pr(** ***X|K*** **) under the aspatial model.** b) Structure results based on the second order rate of change (Δ*K* method) under the aspatial model. For each *K*, 10 independent simulations were performed.
(TIF)

Figure S2 **a) Structure results illustrating changes in ln Pr(** ***X|K*** **) under the spatial model.** b) Structure results based on the second order rate of change (Δ*K* method) under the spatial model. For each *K*, 10 independent simulations were performed.
(TIF)

Table S1 **Genetic diversity statistics per locus and population for** ***Phyllodactylus tuberculosus*** **sampled throughout the Alamos, Sonora region.**
(DOCX)

Table S2 **Multiple regression on distance matrices (MRM) results showing the relationship between pairwise genetic distance (linearized** ***D*** **_est_) and least-cost path cost distances incorporating landscape heterogeneity.** Candidate models tested were based on a priori hypotheses and to minimize collinearity among predictors. Optimal cost values used to parameterize resistance surfaces prior to calculating each least-cost path were selected based on Mantel *r* correlation coefficients. VIF = Variance Inflation Factor.
(DOCX)

Table S3 **Multiple regression on distance matrices (MRM) results showing the relationship between pairwise genetic distance (linearized** ***D*** **_est_) and resistance distances incorporating landscape heterogeneity.** Candidate models tested were based on a priori hypotheses. Optimal cost values used to parameterize resistance surfaces prior to calculating resistance distances were selected based on Mantel *r* correlation coefficients. VIF = Variance Inflation Factor.
(DOCX)
