# Supplementary figures and images for: Landscape Genetics of Leaf-Toed Geckos in the Tropical Dry Forest of Northern Mexico
Source: PLoS One. 2013 Feb 25;8(2):e57433. doi: 10.1371/journal.pone.0057433 (PMC3581464; doi:10.1371/journal.pone.0057433)

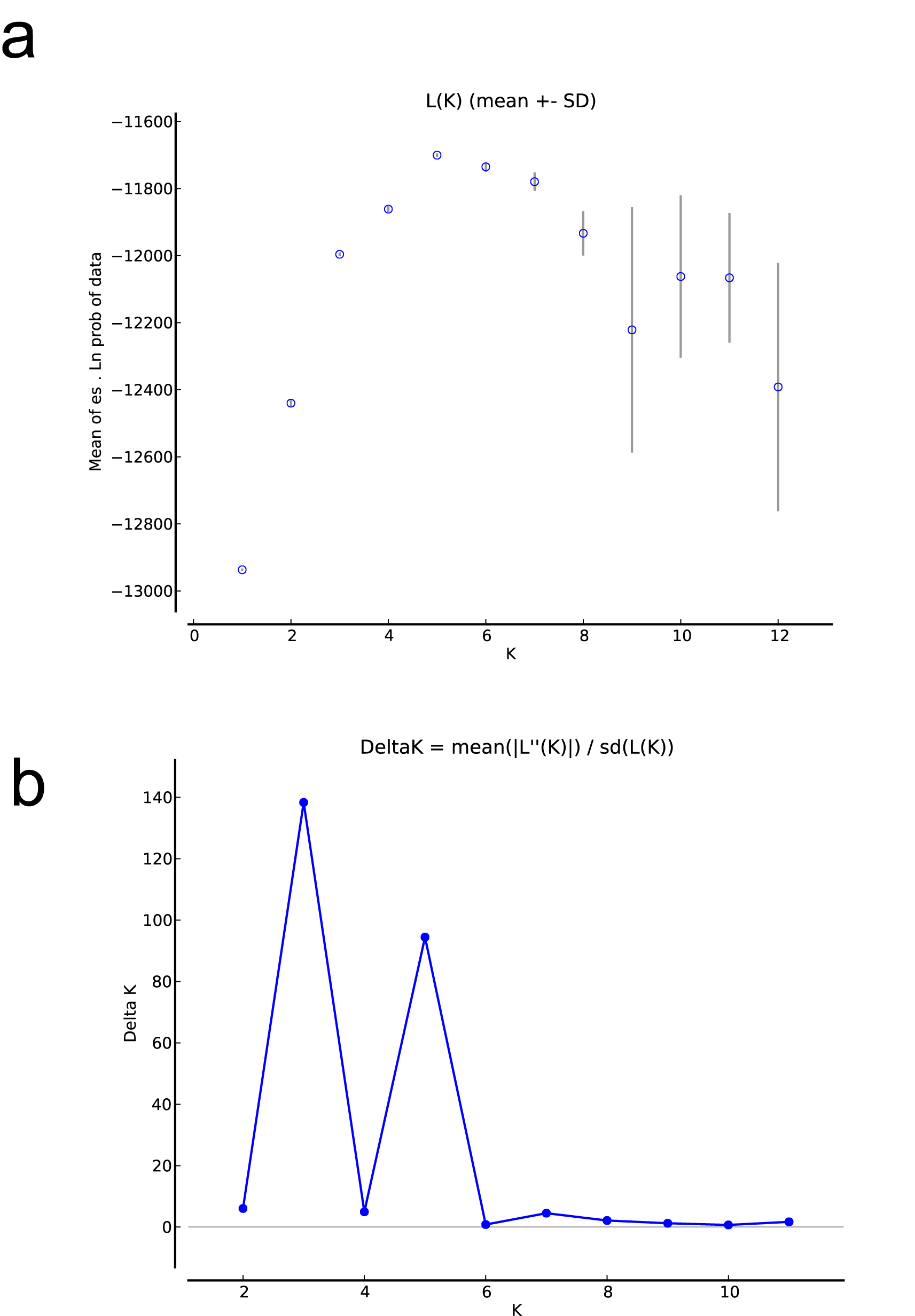

Supplement: Figure S1 — a) Structure results illustrating changes in ln Pr( X|K ) under the aspatial model. b) Structure results based on the second order rate of change (ΔK method) under the aspatial model. For each K, 10 independent simulations were performed. (TIF) [file pone.0057433.s001.tif]

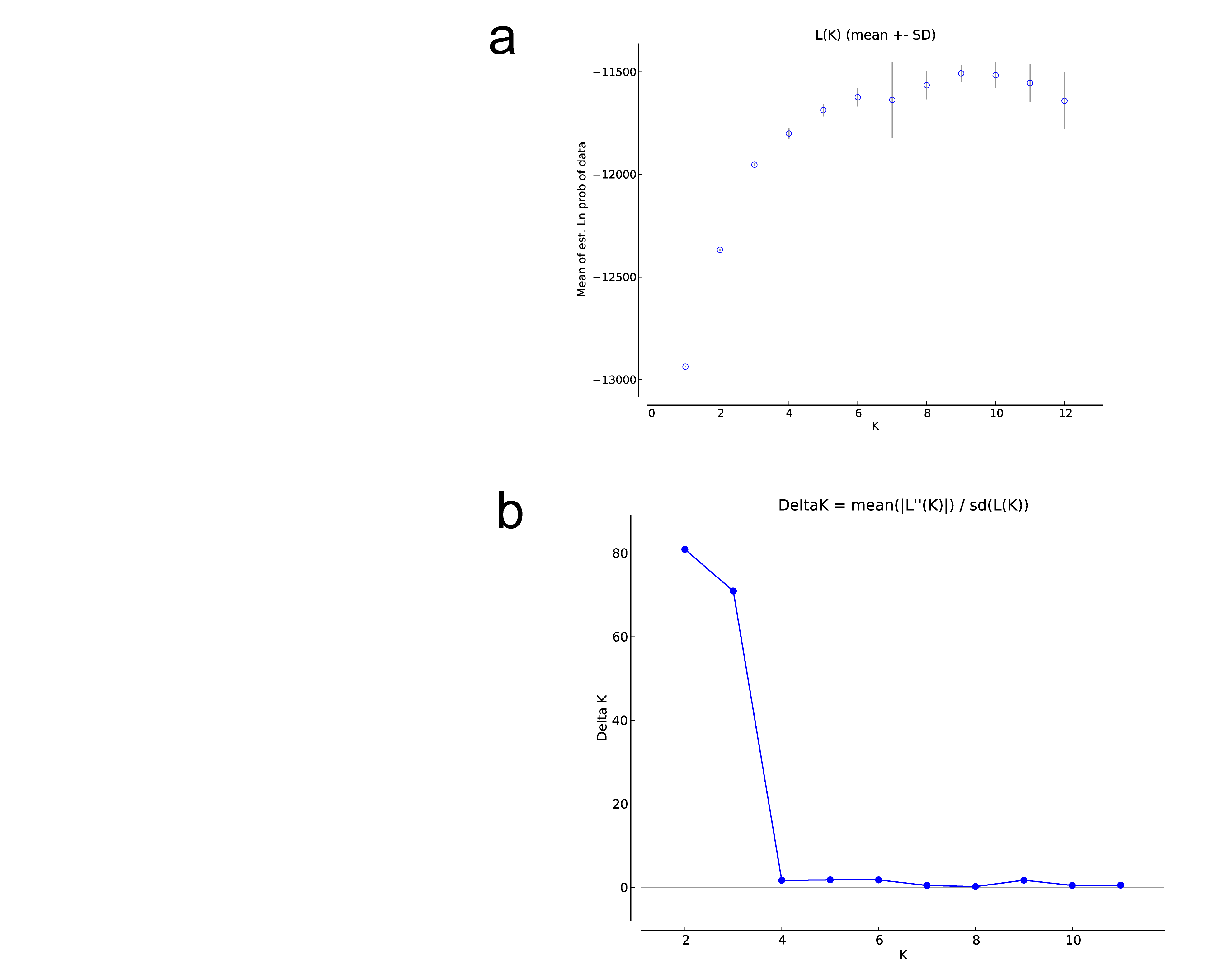

Supplement: Figure S2 — a) Structure results illustrating changes in ln Pr( X|K ) under the spatial model. b) Structure results based on the second order rate of change (ΔK method) under the spatial model. For each K, 10 independent simulations were performed. (TIF) [file pone.0057433.s002.tif]
