# Supplementary material for: Landscape Genetics of Leaf-Toed Geckos in the Tropical Dry Forest of Northern Mexico
Source: PLoS One. 2013 Feb 25;8(2):e57433. doi: 10.1371/journal.pone.0057433 (PMC3581464; doi:10.1371/journal.pone.0057433)
Supplement: Table S1 — Genetic diversity statistics per locus and population for Phyllodactylus tuberculosus sampled throughout the Alamos, Sonora region. (DOCX) [file pone.0057433.s003.docx]

| **Supplementary Table S1** Genetic diversity statistics per locus and population for *Phyllodactylus tuberculosus* sampled throughout the Alamos, Sonora region. | | | | | | | | | | | | |  |  |  |  |  |  |
| --- | --- | --- | --- | --- | --- | --- | --- | --- | --- | --- | --- | --- | --- | --- | --- | --- | --- | --- |
|  |  |  |  |  |  |  |  |  |  |  |  |  |  |  |  |  |  |  |

| Locus | Population | Het obs | Het exp | Het RD | Var | VarRD | Var repeat | Var repeatRD | ShannonID | MinAllele | MeanAllele | MaxAllele | Analys.Chrom. | Num.Alleles | SMM Exp.Num.Alleles | IAM Exp.Num.Alleles | Hs Nei | Fis,Pop |
| --- | --- | --- | --- | --- | --- | --- | --- | --- | --- | --- | --- | --- | --- | --- | --- | --- | --- | --- |
|  |  |  |  |  |  |  |  |  |  |  |  |  |  |  |  |  |  |  |
| G2_22 | Aduana | 0.765 | 0.925 | 0.925 | 289.516 | 289.516 | 32.168 | 32.168 | 2.455 | 231 | 261.618 | 306 | 34 | 14 | 13.186 | 16.711 | 0.914 | 0.163 |
| G2_37 | Aduana | 0.867 | 0.901 | 0.901 | 124.510 | 124.510 | 13.834 | 13.834 | 2.156 | 249 | 268.800 | 285 | 30 | 10 | 10.576 | 13.670 | 0.886 | 0.022 |
| G2_59 | Aduana | 0.294 | 0.540 | 0.540 | 3.947 | 3.947 | 0.439 | 0.439 | 0.872 | 259 | 260.412 | 265 | 34 | 3 | 3.482 | 4.543 | 0.536 | 0.451 |
| G2_85 | Aduana | 0.647 | 0.642 | 0.642 | 12.335 | 12.335 | 0.771 | 0.771 | 1.144 | 243 | 252.294 | 263 | 34 | 5 | 4.239 | 5.884 | 0.632 | -0.024 |
| G2_96 | Aduana | 0.765 | 0.824 | 0.824 | 70.046 | 70.046 | 4.378 | 4.378 | 1.721 | 197 | 214.882 | 225 | 34 | 7 | 7.119 | 10.325 | 0.812 | 0.058 |
| P12 | Aduana | 0.824 | 0.807 | 0.807 | 113.569 | 113.569 | 7.098 | 7.098 | 1.829 | 206 | 227.647 | 254 | 34 | 9 | 6.679 | 9.730 | 0.795 | -0.036 |
| P15 | Aduana | 0.706 | 0.831 | 0.831 | 58.310 | 58.310 | 3.644 | 3.644 | 1.743 | 213 | 224.412 | 241 | 34 | 7 | 7.338 | 10.612 | 0.820 | 0.139 |
| P19 | Aduana | 0.824 | 0.788 | 0.788 | 112.713 | 112.713 | 7.045 | 7.045 | 1.722 | 183 | 204.882 | 219 | 34 | 8 | 6.222 | 9.082 | 0.775 | -0.062 |
| P1 | Aduana | 0.765 | 0.848 | 0.848 | 53.847 | 53.847 | 3.365 | 3.365 | 1.869 | 231 | 244.176 | 259 | 34 | 8 | 7.964 | 11.398 | 0.837 | 0.086 |
| P2 | Aduana | 0.714 | 0.765 | 0.765 | 98.434 | 98.434 | 6.152 | 6.152 | 1.549 | 233 | 244.286 | 261 | 28 | 6 | 5.633 | 7.825 | 0.751 | 0.049 |
| P6 | Aduana | 0.824 | 0.752 | 0.752 | 50.210 | 50.210 | 3.138 | 3.138 | 1.438 | 198 | 206.824 | 222 | 34 | 5 | 5.557 | 8.080 | 0.740 | -0.113 |
| P7 | Aduana | 0.643 | 0.794 | 0.794 | 61.037 | 61.037 | 3.815 | 3.815 | 1.657 | 235 | 253.000 | 267 | 28 | 7 | 6.194 | 8.591 | 0.782 | 0.178 |
|  |  |  |  |  |  |  |  |  |  |  |  |  |  |  |  |  |  |  |
| G2_22 | Alamos | 0.861 | 0.910 | 0.910 | 210.026 | 210.026 | 23.336 | 23.336 | 2.508 | 234 | 260.125 | 285 | 72 | 17 | 12.878 | 21.685 | 0.904 | 0.048 |
| G2_37 | Alamos | 0.889 | 0.915 | 0.915 | 143.533 | 143.533 | 15.948 | 15.948 | 2.517 | 243 | 268.042 | 294 | 72 | 16 | 13.411 | 22.353 | 0.908 | 0.022 |
| G2_59 | Alamos | 0.111 | 0.412 | 0.412 | 4.421 | 4.421 | 0.491 | 0.491 | 0.736 | 259 | 260.125 | 265 | 72 | 3 | 2.984 | 3.852 | 0.411 | 0.730 |
| G2_85 | Alamos | 0.444 | 0.665 | 0.665 | 12.767 | 12.767 | 0.798 | 0.798 | 1.126 | 251 | 254.722 | 271 | 72 | 4 | 4.725 | 7.721 | 0.662 | 0.329 |
| G2_96 | Alamos | 0.528 | 0.764 | 0.764 | 71.011 | 71.011 | 4.438 | 4.438 | 1.561 | 201 | 217.444 | 233 | 72 | 7 | 6.074 | 10.707 | 0.761 | 0.306 |
| P12 | Alamos | 0.914 | 0.836 | 0.836 | 56.411 | 56.411 | 3.526 | 3.526 | 1.854 | 206 | 229.371 | 242 | 70 | 8 | 7.958 | 14.223 | 0.830 | -0.102 |
| P15 | Alamos | 0.800 | 0.790 | 0.790 | 32.666 | 32.666 | 2.042 | 2.042 | 1.633 | 213 | 221.971 | 237 | 70 | 7 | 6.589 | 11.675 | 0.784 | -0.021 |
| P19 | Alamos | 0.771 | 0.808 | 0.808 | 57.361 | 57.361 | 3.585 | 3.585 | 1.760 | 183 | 204.029 | 219 | 70 | 8 | 7.058 | 12.583 | 0.803 | 0.039 |
| P1 | Alamos | 0.788 | 0.869 | 0.869 | 112.459 | 112.459 | 7.029 | 7.029 | 2.117 | 231 | 247.182 | 271 | 66 | 11 | 9.460 | 16.383 | 0.863 | 0.087 |
| P2 | Alamos | 0.583 | 0.753 | 0.753 | 139.690 | 139.690 | 8.731 | 8.731 | 1.594 | 229 | 244.167 | 261 | 72 | 8 | 5.865 | 10.263 | 0.749 | 0.221 |
| P6 | Alamos | 0.882 | 0.766 | 0.766 | 33.068 | 33.068 | 2.067 | 2.067 | 1.576 | 190 | 204.353 | 214 | 68 | 6 | 6.089 | 10.588 | 0.759 | -0.162 |
| P7 | Alamos | 0.625 | 0.813 | 0.813 | 78.821 | 78.821 | 4.926 | 4.926 | 1.800 | 235 | 247.813 | 267 | 64 | 9 | 7.163 | 12.465 | 0.808 | 0.227 |
|  |  |  |  |  |  |  |  |  |  |  |  |  |  |  |  |  |  |  |
| G2_22 | Choquincahui | 0.806 | 0.894 | 0.894 | 106.156 | 106.156 | 11.795 | 11.795 | 2.352 | 231 | 253.500 | 273 | 62 | 14 | 11.148 | 18.376 | 0.888 | 0.091 |
| G2_37 | Choquincahui | 0.893 | 0.895 | 0.895 | 127.064 | 127.064 | 14.118 | 14.118 | 2.415 | 246 | 267.750 | 291 | 56 | 15 | 11.166 | 17.745 | 0.887 | -0.006 |
| G2_59 | Choquincahui | 0.367 | 0.345 | 0.345 | 4.116 | 4.116 | 0.457 | 0.457 | 0.636 | 259 | 264.050 | 265 | 60 | 3 | 2.603 | 3.126 | 0.342 | -0.072 |
| G2_85 | Choquincahui | 0.533 | 0.652 | 0.652 | 11.860 | 11.860 | 0.741 | 0.741 | 1.113 | 251 | 257.933 | 263 | 60 | 4 | 4.543 | 7.080 | 0.647 | 0.176 |
| G2_96 | Choquincahui | 0.467 | 0.723 | 0.723 | 107.656 | 107.656 | 6.729 | 6.729 | 1.552 | 189 | 202.267 | 233 | 60 | 7 | 5.359 | 8.809 | 0.719 | 0.351 |
| P12 | Choquincahui | 0.700 | 0.752 | 0.752 | 63.837 | 63.837 | 3.990 | 3.990 | 1.619 | 222 | 237.600 | 254 | 60 | 8 | 5.801 | 9.703 | 0.746 | 0.062 |
| P15 | Choquincahui | 0.857 | 0.740 | 0.740 | 25.579 | 25.579 | 1.599 | 1.599 | 1.435 | 217 | 226.857 | 237 | 56 | 6 | 5.589 | 9.133 | 0.732 | -0.170 |
| P19 | Choquincahui | 0.806 | 0.804 | 0.804 | 77.424 | 77.424 | 4.839 | 4.839 | 1.831 | 187 | 205.774 | 223 | 62 | 10 | 6.901 | 11.882 | 0.797 | -0.012 |
| P1 | Choquincahui | 0.783 | 0.823 | 0.823 | 233.863 | 233.863 | 14.616 | 14.616 | 1.812 | 231 | 249.783 | 271 | 46 | 9 | 7.324 | 11.585 | 0.814 | 0.039 |
| P2 | Choquincahui | 0.581 | 0.590 | 0.590 | 15.433 | 15.433 | 0.965 | 0.965 | 1.013 | 221 | 233.903 | 237 | 62 | 4 | 4.027 | 5.987 | 0.585 | 0.007 |
| P6 | Choquincahui | 0.767 | 0.718 | 0.718 | 33.247 | 33.247 | 2.078 | 2.078 | 1.504 | 198 | 207.200 | 230 | 60 | 8 | 5.289 | 8.665 | 0.712 | -0.077 |
| P7 | Choquincahui | 0.577 | 0.754 | 0.754 | 32.646 | 32.646 | 2.040 | 2.040 | 1.469 | 231 | 245.538 | 251 | 52 | 6 | 5.790 | 9.357 | 0.748 | 0.229 |
|  |  |  |  |  |  |  |  |  |  |  |  |  |  |  |  |  |  |  |
| G2_22 | CuchujaquiA | 0.767 | 0.912 | 0.912 | 169.472 | 169.472 | 18.830 | 18.830 | 2.477 | 231 | 258.950 | 291 | 60 | 16 | 12.877 | 20.266 | 0.905 | 0.153 |
| G2_37 | CuchujaquiA | 0.833 | 0.914 | 0.914 | 286.464 | 286.464 | 31.829 | 31.829 | 2.528 | 249 | 274.100 | 327 | 60 | 17 | 13.149 | 20.579 | 0.907 | 0.081 |
| G2_59 | CuchujaquiA | 0.333 | 0.447 | 0.447 | 5.947 | 5.947 | 0.661 | 0.661 | 0.768 | 259 | 260.450 | 265 | 60 | 3 | 3.122 | 4.077 | 0.444 | 0.249 |
| G2_85 | CuchujaquiA | 0.833 | 0.691 | 0.691 | 14.151 | 14.151 | 0.884 | 0.884 | 1.299 | 243 | 253.467 | 259 | 60 | 5 | 4.951 | 7.956 | 0.684 | -0.219 |
| G2_96 | CuchujaquiA | 0.733 | 0.759 | 0.759 | 55.046 | 55.046 | 3.440 | 3.440 | 1.730 | 197 | 217.067 | 233 | 60 | 9 | 5.929 | 9.955 | 0.753 | 0.026 |
| P12 | CuchujaquiA | 0.862 | 0.855 | 0.855 | 199.047 | 199.047 | 12.440 | 12.440 | 1.940 | 206 | 228.621 | 250 | 58 | 8 | 8.645 | 14.505 | 0.847 | -0.017 |
| P15 | CuchujaquiA | 0.769 | 0.801 | 0.801 | 51.131 | 51.131 | 3.196 | 3.196 | 1.642 | 213 | 223.077 | 233 | 52 | 6 | 6.760 | 11.080 | 0.793 | 0.030 |
| P19 | CuchujaquiA | 0.750 | 0.854 | 0.854 | 130.909 | 130.909 | 8.182 | 8.182 | 1.967 | 183 | 205.000 | 219 | 56 | 9 | 8.585 | 14.255 | 0.847 | 0.115 |
| P1 | CuchujaquiA | 0.750 | 0.877 | 0.877 | 95.231 | 95.231 | 5.952 | 5.952 | 2.098 | 231 | 245.429 | 271 | 56 | 10 | 9.795 | 15.973 | 0.870 | 0.138 |
| P2 | CuchujaquiA | 0.552 | 0.646 | 0.646 | 127.676 | 127.676 | 7.980 | 7.980 | 1.293 | 229 | 240.793 | 265 | 58 | 6 | 4.477 | 6.900 | 0.641 | 0.140 |
| P6 | CuchujaquiA | 0.586 | 0.623 | 0.623 | 12.472 | 12.472 | 0.779 | 0.779 | 1.155 | 194 | 204.138 | 218 | 58 | 6 | 4.274 | 6.463 | 0.618 | 0.051 |
| P7 | CuchujaquiA | 0.560 | 0.865 | 0.865 | 65.593 | 65.593 | 4.100 | 4.100 | 2.023 | 231 | 245.720 | 263 | 50 | 9 | 9.070 | 14.415 | 0.860 | 0.349 |
|  |  |  |  |  |  |  |  |  |  |  |  |  |  |  |  |  |  |  |
| G2_22 | CuchujaquiB | 0.905 | 0.918 | 0.918 | 175.759 | 175.759 | 19.529 | 19.529 | 2.477 | 231 | 260.893 | 282 | 84 | 14 | 13.927 | 24.350 | 0.912 | 0.008 |
| G2_37 | CuchujaquiB | 0.756 | 0.882 | 0.882 | 199.111 | 199.111 | 22.123 | 22.123 | 2.366 | 249 | 276.000 | 315 | 82 | 15 | 10.320 | 18.987 | 0.877 | 0.138 |
| G2_59 | CuchujaquiB | 0.571 | 0.560 | 0.560 | 5.484 | 5.484 | 0.609 | 0.609 | 0.941 | 259 | 260.714 | 265 | 84 | 3 | 3.895 | 5.910 | 0.557 | -0.026 |
| G2_85 | CuchujaquiB | 0.619 | 0.629 | 0.629 | 10.967 | 10.967 | 0.685 | 0.685 | 1.121 | 243 | 253.143 | 259 | 84 | 5 | 4.416 | 7.178 | 0.625 | 0.009 |
| G2_96 | CuchujaquiB | 0.762 | 0.743 | 0.743 | 43.243 | 43.243 | 2.703 | 2.703 | 1.596 | 197 | 214.286 | 229 | 84 | 8 | 5.743 | 10.360 | 0.739 | -0.031 |
| P12 | CuchujaquiB | 0.829 | 0.836 | 0.836 | 149.410 | 149.410 | 9.338 | 9.338 | 1.943 | 206 | 228.146 | 250 | 82 | 9 | 8.003 | 14.990 | 0.831 | 0.003 |
| P15 | CuchujaquiB | 0.744 | 0.826 | 0.826 | 76.097 | 76.097 | 4.756 | 4.756 | 1.871 | 213 | 222.179 | 241 | 78 | 8 | 7.605 | 14.026 | 0.821 | 0.094 |
| P19 | CuchujaquiB | 0.833 | 0.839 | 0.839 | 89.122 | 89.122 | 5.570 | 5.570 | 1.832 | 183 | 205.714 | 219 | 84 | 7 | 8.118 | 15.314 | 0.834 | 0.001 |
| P1 | CuchujaquiB | 0.810 | 0.867 | 0.867 | 124.225 | 124.225 | 7.764 | 7.764 | 2.106 | 231 | 245.333 | 275 | 84 | 11 | 9.417 | 17.644 | 0.862 | 0.061 |
| P2 | CuchujaquiB | 0.775 | 0.765 | 0.765 | 123.886 | 123.886 | 7.743 | 7.743 | 1.567 | 229 | 238.750 | 265 | 80 | 6 | 6.106 | 11.053 | 0.760 | -0.020 |
| P6 | CuchujaquiB | 0.786 | 0.690 | 0.690 | 12.493 | 12.493 | 0.781 | 0.781 | 1.273 | 198 | 203.810 | 214 | 84 | 5 | 5.027 | 8.668 | 0.685 | -0.146 |
| P7 | CuchujaquiB | 0.744 | 0.838 | 0.838 | 65.835 | 65.835 | 4.115 | 4.115 | 1.906 | 231 | 246.333 | 263 | 78 | 9 | 8.041 | 14.841 | 0.833 | 0.107 |
|  |  |  |  |  |  |  |  |  |  |  |  |  |  |  |  |  |  |  |
| G2_22 | Mocuzari | 0.867 | 0.921 | 0.921 | 143.174 | 143.174 | 15.908 | 15.908 | 2.495 | 237 | 251.750 | 279 | 60 | 15 | 14.047 | 21.581 | 0.914 | 0.051 |
| G2_37 | Mocuzari | 0.793 | 0.826 | 0.826 | 58.141 | 58.141 | 6.460 | 6.460 | 1.890 | 246 | 262.707 | 276 | 58 | 9 | 7.519 | 12.722 | 0.819 | 0.031 |
| G2_59 | Mocuzari | 0.433 | 0.437 | 0.437 | 2.949 | 2.949 | 0.328 | 0.328 | 0.728 | 259 | 260.000 | 265 | 60 | 3 | 3.068 | 3.971 | 0.433 | -0.001 |
| G2_85 | Mocuzari | 0.467 | 0.567 | 0.567 | 12.728 | 12.728 | 0.795 | 0.795 | 1.003 | 247 | 254.467 | 263 | 60 | 4 | 3.855 | 5.590 | 0.563 | 0.172 |
| G2_96 | Mocuzari | 0.833 | 0.855 | 0.855 | 127.928 | 127.928 | 7.995 | 7.995 | 1.917 | 197 | 216.733 | 233 | 60 | 8 | 8.688 | 14.728 | 0.848 | 0.018 |
| P12 | Mocuzari | 0.767 | 0.737 | 0.737 | 36.610 | 36.610 | 2.288 | 2.288 | 1.487 | 222 | 228.000 | 242 | 60 | 6 | 5.565 | 9.230 | 0.731 | -0.049 |
| P15 | Mocuzari | 0.828 | 0.833 | 0.833 | 37.750 | 37.750 | 2.359 | 2.359 | 1.796 | 213 | 223.069 | 237 | 58 | 7 | 7.765 | 13.128 | 0.826 | -0.002 |
| P19 | Mocuzari | 0.759 | 0.760 | 0.760 | 45.595 | 45.595 | 2.850 | 2.850 | 1.544 | 195 | 212.862 | 223 | 58 | 7 | 5.938 | 9.891 | 0.754 | -0.006 |
| P1 | Mocuzari | 0.724 | 0.768 | 0.768 | 74.551 | 74.551 | 4.659 | 4.659 | 1.570 | 227 | 237.897 | 263 | 58 | 7 | 6.073 | 10.149 | 0.761 | 0.049 |
| P2 | Mocuzari | 0.600 | 0.769 | 0.769 | 130.075 | 130.075 | 8.130 | 8.130 | 1.636 | 221 | 235.600 | 261 | 60 | 8 | 6.119 | 10.323 | 0.764 | 0.215 |
| P6 | Mocuzari | 0.533 | 0.507 | 0.507 | 11.656 | 11.656 | 0.729 | 0.729 | 0.900 | 198 | 203.933 | 210 | 60 | 4 | 3.464 | 4.763 | 0.502 | -0.062 |
| P7 | Mocuzari | 0.483 | 0.773 | 0.773 | 48.963 | 48.963 | 3.060 | 3.060 | 1.617 | 231 | 249.138 | 259 | 58 | 7 | 6.179 | 10.350 | 0.769 | 0.372 |
|  |  |  |  |  |  |  |  |  |  |  |  |  |  |  |  |  |  |  |
| G2_22 | Navojoa | 0.900 | 0.926 | 0.926 | 384.063 | 384.063 | 42.674 | 42.674 | 2.250 | 234 | 262.800 | 303 | 20 | 11 | 10.929 | 12.281 | 0.903 | 0.003 |
| G2_37 | Navojoa | 0.800 | 0.900 | 0.900 | 141.158 | 141.158 | 15.684 | 15.684 | 2.042 | 249 | 267.000 | 285 | 20 | 9 | 9.277 | 10.884 | 0.879 | 0.090 |
| G2_59 | Navojoa | 0.300 | 0.426 | 0.426 | 4.050 | 4.050 | 0.450 | 0.450 | 0.731 | 259 | 260.050 | 265 | 20 | 3 | 2.654 | 3.055 | 0.418 | 0.283 |
| G2_85 | Navojoa | 0.700 | 0.521 | 0.521 | 4.168 | 4.168 | 0.261 | 0.261 | 0.688 | 251 | 252.800 | 255 | 20 | 2 | 3.136 | 3.771 | 0.503 | -0.393 |
| G2_96 | Navojoa | 0.500 | 0.658 | 0.658 | 40.884 | 40.884 | 2.555 | 2.555 | 1.106 | 197 | 215.600 | 229 | 20 | 4 | 4.088 | 5.178 | 0.645 | 0.224 |
| P12 | Navojoa | 0.800 | 0.763 | 0.763 | 37.726 | 37.726 | 2.358 | 2.358 | 1.522 | 210 | 227.600 | 238 | 20 | 6 | 5.312 | 6.820 | 0.742 | -0.078 |
| P15 | Navojoa | 0.700 | 0.868 | 0.868 | 84.674 | 84.674 | 5.292 | 5.292 | 1.874 | 213 | 223.600 | 241 | 20 | 8 | 7.860 | 9.587 | 0.850 | 0.176 |
| P19 | Navojoa | 0.800 | 0.805 | 0.805 | 89.095 | 89.095 | 5.568 | 5.568 | 1.670 | 183 | 206.600 | 223 | 20 | 7 | 6.075 | 7.729 | 0.784 | -0.020 |
| P1 | Navojoa | 1.000 | 0.826 | 0.826 | 54.358 | 54.358 | 3.397 | 3.397 | 1.640 | 231 | 242.400 | 255 | 20 | 6 | 6.561 | 8.268 | 0.800 | -0.250 |
| P2 | Navojoa | 0.800 | 0.816 | 0.816 | 116.674 | 116.674 | 7.292 | 7.292 | 1.617 | 233 | 242.400 | 261 | 20 | 6 | 6.307 | 7.990 | 0.795 | -0.007 |
| P6 | Navojoa | 0.700 | 0.695 | 0.695 | 35.537 | 35.537 | 2.221 | 2.221 | 1.469 | 198 | 207.200 | 222 | 20 | 7 | 4.441 | 5.676 | 0.676 | -0.035 |
| P7 | Navojoa | 0.700 | 0.842 | 0.842 | 59.621 | 59.621 | 3.726 | 3.726 | 1.680 | 235 | 248.600 | 259 | 20 | 6 | 6.989 | 8.720 | 0.824 | 0.150 |
|  |  |  |  |  |  |  |  |  |  |  |  |  |  |  |  |  |  |  |
| G2_22 | Quintero | 0.867 | 0.915 | 0.915 | 173.400 | 173.400 | 19.267 | 19.267 | 2.493 | 231 | 253.700 | 291 | 60 | 16 | 13.290 | 20.739 | 0.908 | 0.045 |
| G2_37 | Quintero | 0.867 | 0.901 | 0.901 | 163.271 | 163.271 | 18.141 | 18.141 | 2.327 | 249 | 272.500 | 297 | 60 | 13 | 11.740 | 18.908 | 0.894 | 0.030 |
| G2_59 | Quintero | 0.207 | 0.189 | 0.189 | 3.397 | 3.397 | 0.377 | 0.377 | 0.333 | 259 | 264.379 | 265 | 58 | 2 | 1.864 | 2.001 | 0.187 | -0.107 |
| G2_85 | Quintero | 0.600 | 0.548 | 0.548 | 10.210 | 10.210 | 0.638 | 0.638 | 1.075 | 247 | 255.600 | 263 | 60 | 5 | 3.724 | 5.310 | 0.543 | -0.105 |
| G2_96 | Quintero | 0.464 | 0.768 | 0.768 | 88.140 | 88.140 | 5.509 | 5.509 | 1.707 | 189 | 203.929 | 221 | 56 | 8 | 6.070 | 10.056 | 0.764 | 0.392 |
| P12 | Quintero | 0.724 | 0.690 | 0.690 | 45.633 | 45.633 | 2.852 | 2.852 | 1.458 | 230 | 238.345 | 254 | 58 | 7 | 4.933 | 7.865 | 0.684 | -0.059 |
| P15 | Quintero | 0.667 | 0.803 | 0.803 | 34.260 | 34.260 | 2.141 | 2.141 | 1.659 | 217 | 225.667 | 237 | 60 | 6 | 6.879 | 11.736 | 0.798 | 0.164 |
| P19 | Quintero | 0.767 | 0.858 | 0.858 | 85.080 | 85.080 | 5.318 | 5.318 | 2.090 | 183 | 205.933 | 227 | 60 | 12 | 8.822 | 14.932 | 0.852 | 0.100 |
| P1 | Quintero | 0.875 | 0.855 | 0.855 | 161.702 | 161.702 | 10.106 | 10.106 | 2.007 | 231 | 246.000 | 275 | 48 | 10 | 8.523 | 13.482 | 0.845 | -0.035 |
| P2 | Quintero | 0.586 | 0.520 | 0.520 | 15.022 | 15.022 | 0.939 | 0.939 | 0.797 | 229 | 233.828 | 237 | 58 | 3 | 3.537 | 4.898 | 0.515 | -0.138 |
| P6 | Quintero | 0.724 | 0.745 | 0.745 | 37.537 | 37.537 | 2.346 | 2.346 | 1.602 | 194 | 206.276 | 230 | 58 | 9 | 5.671 | 9.369 | 0.738 | 0.019 |
| P7 | Quintero | 0.333 | 0.712 | 0.712 | 76.705 | 76.705 | 4.794 | 4.794 | 1.452 | 231 | 239.200 | 259 | 60 | 6 | 5.207 | 8.494 | 0.709 | 0.530 |
|  |  |  |  |  |  |  |  |  |  |  |  |  |  |  |  |  |  |  |
| G2_22 | SanAntonio | 0.933 | 0.903 | 0.903 | 114.921 | 114.921 | 12.769 | 12.769 | 2.219 | 240 | 263.100 | 282 | 30 | 11 | 10.747 | 13.831 | 0.887 | -0.052 |
| G2_37 | SanAntonio | 0.923 | 0.902 | 0.902 | 176.178 | 176.178 | 19.575 | 19.575 | 2.185 | 243 | 267.462 | 297 | 26 | 11 | 10.210 | 12.697 | 0.883 | -0.045 |
| G2_59 | SanAntonio | 0.250 | 0.366 | 0.366 | 3.505 | 3.505 | 0.389 | 0.389 | 0.652 | 259 | 259.875 | 265 | 24 | 3 | 2.444 | 2.775 | 0.361 | 0.307 |
| G2_85 | SanAntonio | 0.615 | 0.588 | 0.588 | 6.326 | 6.326 | 0.395 | 0.395 | 0.908 | 247 | 252.385 | 255 | 26 | 3 | 3.684 | 4.745 | 0.575 | -0.070 |
| G2_96 | SanAntonio | 0.786 | 0.825 | 0.825 | 79.471 | 79.471 | 4.967 | 4.967 | 1.738 | 197 | 211.714 | 225 | 28 | 7 | 6.982 | 9.592 | 0.811 | 0.031 |
| P12 | SanAntonio | 0.786 | 0.833 | 0.833 | 57.905 | 57.905 | 3.619 | 3.619 | 1.804 | 222 | 235.143 | 254 | 28 | 8 | 7.219 | 9.876 | 0.819 | 0.040 |
| P15 | SanAntonio | 0.600 | 0.784 | 0.784 | 33.030 | 33.030 | 2.064 | 2.064 | 1.633 | 209 | 221.267 | 233 | 30 | 7 | 6.047 | 8.547 | 0.774 | 0.224 |
| P19 | SanAntonio | 0.846 | 0.794 | 0.794 | 166.400 | 166.400 | 10.400 | 10.400 | 1.580 | 183 | 199.000 | 219 | 26 | 6 | 6.129 | 8.343 | 0.777 | -0.089 |
| P1 | SanAntonio | 0.769 | 0.862 | 0.862 | 97.698 | 97.698 | 6.106 | 6.106 | 1.832 | 231 | 242.538 | 259 | 26 | 7 | 8.116 | 10.649 | 0.846 | 0.091 |
| P2 | SanAntonio | 0.800 | 0.736 | 0.736 | 54.510 | 54.510 | 3.407 | 3.407 | 1.441 | 233 | 241.800 | 261 | 30 | 6 | 5.227 | 7.350 | 0.722 | -0.108 |
| P6 | SanAntonio | 0.846 | 0.751 | 0.751 | 45.095 | 45.095 | 2.818 | 2.818 | 1.568 | 194 | 203.846 | 226 | 26 | 7 | 5.352 | 7.298 | 0.734 | -0.153 |
| P7 | SanAntonio | 0.733 | 0.853 | 0.853 | 67.476 | 67.476 | 4.217 | 4.217 | 1.881 | 235 | 252.200 | 267 | 30 | 8 | 7.985 | 10.986 | 0.840 | 0.127 |
|  |  |  |  |  |  |  |  |  |  |  |  |  |  |  |  |  |  |  |
| G2_22 | Sierrita | 0.737 | 0.902 | 0.902 | 174.499 | 174.499 | 19.389 | 19.389 | 2.497 | 231 | 259.184 | 288 | 76 | 17 | 12.043 | 21.001 | 0.898 | 0.179 |
| G2_37 | Sierrita | 0.941 | 0.921 | 0.921 | 110.165 | 110.165 | 12.241 | 12.241 | 2.501 | 243 | 267.882 | 288 | 68 | 15 | 14.222 | 22.835 | 0.914 | -0.030 |
| G2_59 | Sierrita | 0.323 | 0.288 | 0.288 | 3.522 | 3.522 | 0.391 | 0.391 | 0.550 | 259 | 259.774 | 265 | 62 | 3 | 2.335 | 2.689 | 0.285 | -0.132 |
| G2_85 | Sierrita | 0.351 | 0.442 | 0.442 | 25.582 | 25.582 | 1.599 | 1.599 | 0.871 | 247 | 251.703 | 271 | 74 | 5 | 3.153 | 4.196 | 0.440 | 0.202 |
| G2_96 | Sierrita | 0.553 | 0.720 | 0.720 | 26.184 | 26.184 | 1.636 | 1.636 | 1.426 | 197 | 219.947 | 229 | 76 | 6 | 5.383 | 9.320 | 0.717 | 0.229 |
| P12 | Sierrita | 0.789 | 0.778 | 0.778 | 97.269 | 97.269 | 6.079 | 6.079 | 1.723 | 206 | 226.105 | 258 | 76 | 8 | 6.359 | 11.452 | 0.773 | -0.021 |
| P15 | Sierrita | 0.622 | 0.854 | 0.854 | 64.690 | 64.690 | 4.043 | 4.043 | 1.954 | 209 | 222.459 | 241 | 74 | 9 | 8.729 | 15.806 | 0.850 | 0.269 |
| P19 | Sierrita | 0.868 | 0.759 | 0.759 | 55.556 | 55.556 | 3.472 | 3.472 | 1.610 | 183 | 205.737 | 219 | 76 | 8 | 5.980 | 10.645 | 0.753 | -0.154 |
| P1 | Sierrita | 0.649 | 0.777 | 0.777 | 125.891 | 125.891 | 7.868 | 7.868 | 1.723 | 231 | 241.162 | 271 | 74 | 8 | 6.337 | 11.330 | 0.773 | 0.161 |
| P2 | Sierrita | 0.676 | 0.619 | 0.619 | 108.167 | 108.167 | 6.760 | 6.760 | 1.375 | 229 | 253.324 | 265 | 74 | 9 | 4.309 | 6.786 | 0.615 | -0.099 |
| P6 | Sierrita | 0.703 | 0.731 | 0.731 | 30.578 | 30.578 | 1.911 | 1.911 | 1.547 | 198 | 205.676 | 222 | 74 | 7 | 5.525 | 9.585 | 0.726 | 0.032 |
| P7 | Sierrita | 0.568 | 0.743 | 0.743 | 92.683 | 92.683 | 5.793 | 5.793 | 1.484 | 227 | 244.405 | 267 | 74 | 6 | 5.712 | 9.997 | 0.739 | 0.232 |
|  |  |  |  |  |  |  |  |  |  |  |  |  |  |  |  |  |  |  |
| G2_22 | TabeloA | 0.815 | 0.863 | 0.863 | 164.038 | 164.038 | 18.226 | 18.226 | 1.990 | 234 | 251.000 | 267 | 54 | 9 | 9.004 | 14.693 | 0.855 | 0.047 |
| G2_37 | TabeloA | 0.962 | 0.881 | 0.881 | 158.783 | 158.783 | 17.643 | 17.643 | 2.190 | 252 | 268.038 | 309 | 52 | 12 | 10.010 | 15.852 | 0.871 | -0.103 |
| G2_59 | TabeloA | 0.185 | 0.539 | 0.539 | 3.283 | 3.283 | 0.365 | 0.365 | 0.899 | 259 | 261.667 | 265 | 54 | 3 | 3.633 | 5.060 | 0.537 | 0.655 |
| G2_85 | TabeloA | 0.444 | 0.469 | 0.469 | 7.894 | 7.894 | 0.493 | 0.493 | 0.889 | 247 | 254.259 | 263 | 54 | 5 | 3.214 | 4.224 | 0.465 | 0.044 |
| G2_96 | TabeloA | 0.593 | 0.818 | 0.818 | 37.283 | 37.283 | 2.330 | 2.330 | 1.695 | 213 | 223.000 | 233 | 54 | 6 | 7.234 | 11.994 | 0.812 | 0.270 |
| P12 | TabeloA | 0.593 | 0.665 | 0.665 | 25.610 | 25.610 | 1.601 | 1.601 | 1.243 | 218 | 225.778 | 238 | 54 | 5 | 4.634 | 7.143 | 0.659 | 0.101 |
| P15 | TabeloA | 0.556 | 0.868 | 0.868 | 122.477 | 122.477 | 7.655 | 7.655 | 2.015 | 201 | 220.704 | 241 | 54 | 9 | 9.263 | 15.054 | 0.863 | 0.356 |
| P19 | TabeloA | 0.852 | 0.854 | 0.854 | 97.459 | 97.459 | 6.091 | 6.091 | 1.983 | 183 | 208.556 | 223 | 54 | 9 | 8.568 | 14.064 | 0.846 | -0.007 |
| P1 | TabeloA | 0.600 | 0.794 | 0.794 | 132.186 | 132.186 | 8.262 | 8.262 | 1.630 | 227 | 242.760 | 275 | 50 | 7 | 6.582 | 10.661 | 0.788 | 0.239 |
| P2 | TabeloA | 0.538 | 0.480 | 0.480 | 16.863 | 16.863 | 1.054 | 1.054 | 1.015 | 229 | 238.000 | 257 | 52 | 6 | 3.268 | 4.316 | 0.475 | -0.133 |
| P6 | TabeloA | 0.792 | 0.730 | 0.730 | 16.312 | 16.312 | 1.020 | 1.020 | 1.364 | 198 | 203.833 | 214 | 48 | 5 | 5.387 | 8.443 | 0.722 | -0.096 |
| P7 | TabeloA | 0.545 | 0.837 | 0.837 | 76.888 | 76.888 | 4.805 | 4.805 | 1.747 | 235 | 245.364 | 259 | 44 | 6 | 7.765 | 12.072 | 0.831 | 0.344 |
|  |  |  |  |  |  |  |  |  |  |  |  |  |  |  |  |  |  |  |
| G2_22 | TabeloB | 0.833 | 0.811 | 0.811 | 176.225 | 176.225 | 19.581 | 19.581 | 1.837 | 234 | 254.250 | 288 | 60 | 10 | 7.077 | 12.088 | 0.804 | -0.037 |
| G2_37 | TabeloB | 0.633 | 0.853 | 0.853 | 144.508 | 144.508 | 16.056 | 16.056 | 2.079 | 252 | 268.000 | 312 | 60 | 12 | 8.584 | 14.569 | 0.848 | 0.253 |
| G2_59 | TabeloB | 0.233 | 0.569 | 0.569 | 3.445 | 3.445 | 0.383 | 0.383 | 0.891 | 259 | 260.750 | 265 | 60 | 3 | 3.867 | 5.616 | 0.567 | 0.588 |
| G2_85 | TabeloB | 0.600 | 0.520 | 0.520 | 8.945 | 8.945 | 0.559 | 0.559 | 0.958 | 243 | 254.267 | 263 | 60 | 5 | 3.543 | 4.928 | 0.515 | -0.166 |
| G2_96 | TabeloB | 0.600 | 0.764 | 0.764 | 52.515 | 52.515 | 3.282 | 3.282 | 1.589 | 201 | 220.400 | 229 | 60 | 6 | 6.022 | 10.136 | 0.759 | 0.210 |
| P12 | TabeloB | 0.536 | 0.687 | 0.687 | 24.431 | 24.431 | 1.527 | 1.527 | 1.294 | 218 | 225.071 | 238 | 56 | 5 | 4.885 | 7.713 | 0.682 | 0.215 |
| P15 | TabeloB | 0.750 | 0.742 | 0.742 | 21.564 | 21.564 | 1.348 | 1.348 | 1.491 | 213 | 221.500 | 237 | 56 | 7 | 5.609 | 9.173 | 0.735 | -0.021 |
| P19 | TabeloB | 0.852 | 0.857 | 0.857 | 84.176 | 84.176 | 5.261 | 5.261 | 1.890 | 195 | 210.778 | 223 | 54 | 7 | 8.729 | 14.300 | 0.849 | -0.003 |
| P1 | TabeloB | 0.833 | 0.813 | 0.813 | 103.209 | 103.209 | 6.451 | 6.451 | 1.800 | 227 | 240.667 | 275 | 60 | 8 | 7.141 | 12.200 | 0.806 | -0.034 |
| P2 | TabeloB | 0.481 | 0.713 | 0.713 | 29.076 | 29.076 | 1.817 | 1.817 | 1.426 | 229 | 239.593 | 257 | 54 | 6 | 5.195 | 8.285 | 0.709 | 0.321 |
| P6 | TabeloB | 0.800 | 0.775 | 0.775 | 23.553 | 23.553 | 1.472 | 1.472 | 1.525 | 194 | 203.800 | 214 | 60 | 6 | 6.231 | 10.537 | 0.768 | -0.041 |
| P7 | TabeloB | 0.538 | 0.868 | 0.868 | 118.805 | 118.805 | 7.425 | 7.425 | 2.009 | 235 | 248.692 | 267 | 52 | 9 | 9.242 | 14.839 | 0.863 | 0.376 |
