# Supplementary material for: Landscape Genetics of Leaf-Toed Geckos in the Tropical Dry Forest of Northern Mexico
Source: PLoS One. 2013 Feb 25;8(2):e57433. doi: 10.1371/journal.pone.0057433 (PMC3581464; doi:10.1371/journal.pone.0057433)
Supplement: Table S2 — Multiple regression on distance matrices (MRM) results showing the relationship between pairwise genetic distance (linearized D est) and least-cost path cost distances incorporating landscape heterogeneity. Candidate models tested were based on a priori hypotheses and to minimize collinearity among predictors. Optimal cost values used to parameterize resistance surfaces prior to calculating each least-cost path were selected based on Mantel r correlation coefficients. VIF = Variance Inflation Factor. (DOCX) [file pone.0057433.s004.docx]

| **Supplementary Table S2** Multiple regression on distance matrices (MRM) results showing the relationship between pairwise genetic distance (linearized *D*_est_) and least-cost path cost distances incorporating landscape heterogeneity. Candidate models tested were based on a priori hypotheses and to minimize collinearity among predictors. Optimal cost values used to parameterize resistance surfaces prior to calculating each least-cost path were selected based on Mantel *r* correlation coefficients. VIF=Variance Inflation Factor. | | | | | | | | | |
| --- | --- | --- | --- | --- | --- | --- | --- | --- | --- |
|  |  |  |  |  |  |  |  |  |  |
|  |  |  |  |  |  |  |  |  |  |
|  |  |  |  |  |  |  |  |  |  |
|  |  |  |  |  |  |  |  |  |  |
| Model | Variables | β | *P* | Model *R*^2^ | *P* | VIF | Model AICc | ΔAICc | Akaike Weight (*w_i_*) |
| A | Euclidean | 2.75E-05 | 0.0014 | 0.621 | 0.0001 | **20.26** | -118.13 | 0.00 | 0.856 |
|  | Anthropogenic | -1.72E-06 | 0.0077 |  |  | 4.20 |  |  |  |
|  | Stream | -6.77E-06 | 0.1152 |  |  | **13.64** |  |  |  |
|  |  |  |  |  |  |  |  |  |  |
| B | Euclidean | 1.62E-05 | 0.0002 | 0.573 | 0.0001 | 3.86 | -112.60 | 5.53 | 0.054 |
|  | Anthropogenic | -1.46E-06 | 0.0426 |  |  | 3.86 |  |  |  |
|  |  |  |  |  |  |  |  |  |  |
| C | Temperature | 1.99E-06 | 0.0052 | 0.566 | 0.0001 | 5.39 | -111.53 | 6.60 | 0.032 |
|  | Stream | -2.65E-06 | 0.3308 |  |  | 5.39 |  |  |  |
|  |  |  |  |  |  |  |  |  |  |
| D | Temperature | 1.44E-06 | 0.0001 | 0.548 | 0.0001 |  | -111.06 | 7.07 | 0.025 |
|  |  |  |  |  |  |  |  |  |  |
| E | Slope | 6.08E-06 | 0.0002 | 0.539 | 0.0002 |  | -109.80 | 8.33 | 0.013 |
|  |  |  |  |  |  |  |  |  |  |
| F | Temperature | 1.58E-06 | 0.0367 | 0.549 | 0.0002 | 6.34 | -108.95 | 9.18 | 0.009 |
|  | Euclidean | -1.04E-06 | 0.827 |  |  | 6.34 |  |  |  |
|  |  |  |  |  |  |  |  |  |  |
| G | Slope | 7.29E-06 | 0.0042 | 0.545 | 0.0002 | 4.44 | -108.41 | 9.72 | 0.007 |
|  | Stream | -1.38E-06 | 0.5563 |  |  | 4.44 |  |  |  |
|  |  |  |  |  |  |  |  |  |  |
| H | Slope | 5.36E-06 | 0.0275 | 0.541 | 0.0004 | 4.31 | -107.86 | 10.25 | 0.005 |
|  | Euclidean | 1.27E-06 | 0.7208 |  |  | 4.31 |  |  |  |
|  |  |  |  |  |  |  |  |  |  |
| I | Euclidean | 8.48E-06 | 0.0002 | 0.444 | 0.0002 |  | -97.46 | 20.67 | 0.000 |
|  |  |  |  |  |  |  |  |  |  |
| J | Forest | 8.09E-06 | 0.0001 | 0.440 | 0.0001 |  | -96.96 | 21.17 | 0.000 |
|  |  |  |  |  |  |  |  |  |  |
| K | Euclidean | 1.31E-05 | 0.0797 | 0.455 | 0.0010 | **12.52** | -96.56 | 21.57 | 0.000 |
|  | Stream | -3.16E-06 | 0.4749 |  |  | **12.52** |  |  |  |
|  |  |  |  |  |  |  |  |  |  |
| L | Euclidean | 1.78E-05 | 0.4923 | 0.446 | 0.0005 | **339.12** | -95.38 | 22.75 | 0.000 |
|  | Forest | -8.94E-06 | 0.7195 |  |  | **339.12** |  |  |  |
|  |  |  |  |  |  |  |  |  |  |
| M | Euclidean | 2.01E-05 | 0.4645 | 0.456 | 0.0019 | **342.20** | -94.32 | 23.81 | 0.000 |
|  | Forest | -6.85E-06 | 0.7767 |  |  | **341.87** |  |  |  |
|  | Stream | -3.08E-06 | 0.4749 |  |  | 12.62 |  |  |  |
|  |  |  |  |  |  |  |  |  |  |
| N | Stream | 5.09E-06 | 0.0005 | 0.371 | 0.0005 |  | -89.30 | 28.83 | 0.000 |
|  |  |  |  |  |  |  |  |  |  |
| O | Anthropogenic | 8.06E-07 | 0.0522 | 0.153 | 0.0522 |  | -69.64 | 48.49 | 0.000 |
