# Supplementary material for: Landscape Genetics of Leaf-Toed Geckos in the Tropical Dry Forest of Northern Mexico
Source: PLoS One. 2013 Feb 25;8(2):e57433. doi: 10.1371/journal.pone.0057433 (PMC3581464; doi:10.1371/journal.pone.0057433)
Supplement: Table S3 — Multiple regression on distance matrices (MRM) results showing the relationship between pairwise genetic distance (linearized D est) and resistance distances incorporating landscape heterogeneity. Candidate models tested were based on a priori hypotheses. Optimal cost values used to parameterize resistance surfaces prior to calculating resistance distances were selected based on Mantel r correlation coefficients. VIF = Variance Inflation Factor. (DOCX) [file pone.0057433.s005.docx]

| **Supplementary Table S3** Multiple regression on distance matrices (MRM) results showing the relationship between pairwise genetic distance (linearized *D*_est_) and resistance distances incorporating landscape heterogeneity. Candidate models tested were based on a priori hypotheses. Optimal cost values used to parameterize resistance surfaces prior to calculating resistance distances were selected based on Mantel *r* correlation coefficients. VIF=Variance Inflation Factor. | | | | | | | | | |
| --- | --- | --- | --- | --- | --- | --- | --- | --- | --- |
|  |  |  |  |  |  |  |  |  |  |
|  |  |  |  |  |  |  |  |  |  |
|  |  |  |  |  |  |  |  |  |  |
|  |  |  |  |  |  |  |  |  |  |
| Model | Variables | β | *P* | Model *R*^2^ | *P* | VIF | Model AICc | ΔAICc | Akaike Weight (*wi*) |
| A | Euclidean | 7.81E-06 | 0.0005 | 0.626 | 0.0001 | 1.65 | -118.94 | 0 | 0.545 |
|  | Slope | 9.00E-02 | 0.0070 |  |  | 1.77 |  |  |  |
|  | Stream | -1.95E-01 | 0.0734 |  |  | 2.11 |  |  |  |
|  |  |  |  |  |  |  |  |  |  |
| B | Euclidean | 8.47E-06 | 0.0006 | 0.654 | 0.0003 | 2.18 | -116.57 | 2.37 | 0.167 |
|  | Anthropogenic | -2.03E-02 | 0.4354 |  |  | 3.31 |  |  |  |
|  | Stream | -2.02E-01 | 0.1782 |  |  | 3.33 |  |  |  |
|  | Forest | 8.64E-02 | 0.7342 |  |  | 4.62 |  |  |  |
|  | Slope | 4.74E-02 | 0.4369 |  |  | 6.48 |  |  |  |
|  | Temperature | 2.75E-02 | 0.5779 |  |  | 8.30 |  |  |  |
|  |  |  |  |  |  |  |  |  |  |
| C | Euclidean | 7.90E-06 | 0.0006 | 0.626 | 0.0002 | 1.92 | -116.56 | 2.38 | 0.166 |
|  | Forest | -1.83E-02 | 0.9152 |  |  | 2.52 |  |  |  |
|  | Stream | -1.87E-01 | 0.1851 |  |  | 3.21 |  |  |  |
|  | Slope | 8.92E-02 | 0.0111 |  |  | 1.88 |  |  |  |
|  |  |  |  |  |  |  |  |  |  |
| D | Euclidean | 8.32E-06 | 0.0003 | 0.606 | 0.0001 | 2.06 | -115.53 | 3.41 | 0.099 |
|  | Slope | 5.05E-02 | 0.0670 |  |  | 1.47 |  |  |  |
|  | Anthropogenic | -2.53E-02 | 0.1106 |  |  | 1.54 |  |  |  |
|  |  |  |  |  |  |  |  |  |  |
| E | Euclidean | 7.52E-06 | 0.0010 | 0.585 | 0.0003 | 1.90 | -112.12 | 6.82 | 0.018 |
|  | Slope | 6.57E-02 | 0.0254 |  |  | 1.36 |  |  |  |
|  | Forest | -1.67E-01 | 0.2163 |  |  | 1.65 |  |  |  |
|  |  |  |  |  |  |  |  |  |  |
| F | Euclidean | 1.10E-05 | 0.0002 | 0.538 | 0.0002 | 1.41 | -107.42 | 11.52 | 0.002 |
|  | Anthropogenic | -3.40E-02 | 0.0437 |  |  | 1.41 |  |  |  |
|  |  |  |  |  |  |  |  |  |  |
| G | Euclidean | 5.75E-06 | 0.0112 | 0.536 | 0.0001 | 1.50 | -107.14 | 11.8 | 0.001 |
|  | Temperature | 4.23E-02 | 0.0385 |  |  | 1.50 |  |  |  |
|  |  |  |  |  |  |  |  |  |  |
| H | Euclidean | 1.04E-05 | 0.0003 | 0.546 | 0.0002 | 1.66 | -106.25 | 12.69 | 0.001 |
|  | Anthropogenic | -4.25E-02 | 0.0868 |  |  | 2.44 |  |  |  |
|  | Forest | 0.119837069 | 0.5310 |  |  | 2.83 |  |  |  |
|  |  |  |  |  |  |  |  |  |  |
| I | Euclidean | 1.10E-05 | 0.0003 | 0.538 | 0.0002 | 1.84 | -105.07 | 13.87 | 0.001 |
|  | Anthropogenic | -3.40E-02 | 0.0597 |  |  | 1.47 |  |  |  |
|  | Stream | 1.87E-03 | 0.9890 |  |  | 1.67 |  |  |  |
|  |  |  |  |  |  |  |  |  |  |
| J | Euclidean | 1.07E-05 | 0.0002 | 0.550 | 0.0003 | 1.86 | -104.33 | 14.61 | 0.000 |
|  | Forest | 1.73E-01 | 0.4801 |  |  | 4.17 |  |  |  |
|  | Stream | -4.75E-02 | 0.7229 |  |  | 2.46 |  |  |  |
|  | Anthropogenic | -4.47E-02 | 0.0786 |  |  | 2.59 |  |  |  |
|  |  |  |  |  |  |  |  |  |  |
| K | Euclidean | 8.48E-06 | 0.0001 | 0.444 | 0.0001 |  | -97.46 | 21.48 | 0.000 |
|  |  |  |  |  |  |  |  |  |  |
| L | Euclidean | 9.81E-06 | 0.0008 | 0.461 | 0.0004 | 1.64 | -97.24 | 21.7 | 0.000 |
|  | Forest | -1.32E-01 | 0.3719 |  |  | 1.64 |  |  |  |
|  |  |  |  |  |  |  |  |  |  |
| M | Euclidean | 9.08E-06 | 0.0004 | 0.448 | 0.0001 | 1.60 | -95.63 | 23.31 | 0.000 |
|  | Stream | -3.98E-02 | 0.7149 |  |  | 1.60 |  |  |  |
|  |  |  |  |  |  |  |  |  |  |
| N | Euclidean | 9.74E-06 | 0.0006 | 0.461 | 0.0004 | 1.79 | -94.92 | 24.02 | 0.000 |
|  | Forest | -1.41E-01 | 0.4670 |  |  | 2.37 |  |  |  |
|  | Stream | 1.09E-02 | 0.9420 |  |  | 2.31 |  |  |  |
|  |  |  |  |  |  |  |  |  |  |
| O | Temp | 0.072 | 0.0013 | 0.400 | 0.0013 |  | -92.42 | 26.52 | 0.000 |
|  |  |  |  |  |  |  |  |  |  |
| P | Slope | 0.101 | 0.0017 | 0.395 | 0.0017 |  | -91.83 | 27.11 | 0.000 |
|  |  |  |  |  |  |  |  |  |  |
| Q | Stream | 0.185 | 0.0412 | 0.129 | 0.0412 |  | -67.82 | 51.12 | 0.000 |
|  |  |  |  |  |  |  |  |  |  |
| R | Forest | 0.247 | 0.0520 | 0.098 | 0.0520 |  | -65.53 | 53.41 | 0.000 |
|  |  |  |  |  |  |  |  |  |  |
| S | Anthropogenic | 0.009 | 0.6197 | 0.010 | 0.6197 |  | -59.39 | 59.55 | 0.000 |
